# Silver-*N*-Heterocyclic Complexes Against *Leishmania major*: In Vitro, In Vivo and In Silico Therapeutic Activities

**DOI:** 10.3390/ph19030356

**Published:** 2026-02-25

**Authors:** Neslihan Şahin, Zübeyda Akın Polat, Derya Gül Gülpınar, Ahmet Duran Ataş, Elvan Üstün, İsmail Özdemir, David Sémeril

**Affiliations:** 1 Department of Engineering Basic Sciences, Faculty of Engineering and Natural Sciences, Malatya Turgut Özal University, 44900 Malatya, Türkiye; neslihan@cumhuriyet.edu.tr; 2 Department of Science Education, Faculty of Education, Cumhuriyet University, 58040 Sivas, Türkiye; 3 Department of Medical Parasitology, School of Medicine, Cumhuriyet University, 58140 Sivas, Türkiye; zakinpolat@cumhuriyet.edu.tr (Z.A.P.); deryagulsagci@gmail.com (D.G.G.); ahmetduranatas@gmail.com (A.D.A.); 4 Department of Chemistry, Faculty of Art and Science, Ordu University, 52200 Ordu, Türkiye; 5 Department of Chemistry, Faculty of Art and Science, İnönü University, 44280 Malatya, Türkiye; ismail.ozdemir@inonu.edu.tr; 6 Synthèse Organométallique et Catalyse, UMR-CNRS 7177, Strasbourg University, 67008 Strasbourg, France

**Keywords:** cutaneous leishmaniasis, in vivo, in vitro, in silico, *N*-heterocyclic carbene, silver complex, molecular docking

## Abstract

**Background**/**Objectives**: Cutaneous leishmaniasis (CL) is a prevalent vector-borne disease characterized by a broad spectrum of clinical manifestations resulting from protozoan parasites belonging to the genus *Leishmania*. The challenges associated with the treatment of CL are attributable to various factors, including but not limited to: drug resistance, the adverse effects of conventional therapeutic interventions and the imperative for novel therapeutic alternatives to address the global health burden posed by this neglected tropical disease. **Methods**: In this study, The therapeutic efficacy of two silver(I)-*N*-heterocyclic carbene (NHC) complexes, namely chloro[1-methallyl-3-(2,4,6-trimethylbenzyl)-5,6-dimethylbenzimidazole-2-ylidene]silver(I) (**2a**) and chloro[1-methallyl-3-(4-chlorobenzyl)-5,6-dimethylbenzimidazole-2-ylidene]silver(I) (**2b**), was evaluated against promastigotes in vitro and in vivo in an experimentally induced CL model in Balb/c mice. **Results**: The findings of this study indicated that these compounds possess the potential to function as effective therapeutic agents, particularly in the treatment of CL. Subsequently, the silver(I) complexes were analyzed by means of molecular docking against LaGP63, LaARG, *N*-myristoyltransferase and farnesyl pyrophosphate synthase. **Conclusions**: According to the docking evaluations, complex **2a** emerged as the most notable molecule in terms of its potential antileishmanial activity.

## 1. Introduction

Leishmaniasis, an affliction caused by protozoan parasites of the genus *Leishmania*, is a disease of significant public health concern. It persists as a prominent vector-borne disease, exerting considerable global public health implications. The clinical manifestations of the disease range from self-resolving cutaneous lesions to severe and potentially fatal visceral forms [1,2,3]. Of these, cutaneous leishmaniasis (CL) is the most prevalent and imposes a significant burden on healthcare systems and affected populations. According to the World Health Organization (WHO), around 95% of CL cases occur in the Americas, the Mediterranean basin, the Middle East and Central Asia. It is estimated that between 600,000 and 1 million new cases occur worldwide each year, but only around 200,000 of these are reported to the WHO, suggesting that the disease is substantially underreported [4,5,6].

Clinically, CL is characterized by skin lesions resulting from the proliferation of *Leishmania* parasites within dermal tissue. The disease manifests with significant variability, presenting as lesions ranging from localized papules to extensive ulcerative lesions. While not necessarily life-threatening, CL can result in significant disfigurement, discomfort and psychological distress, underscoring the necessity for effective treatment strategies [1,2]. Historically, treatment has relied on pentavalent antimonials such as sodium stibogluconate and meglumine antimoniate (MA) [7,8]. However, the emergence of drug resistance, the occurrence of significant adverse effects and the necessity of prolonged treatment regimens underscore the pressing need for alternative therapeutic approaches. Furthermore, the heterogeneity of *Leishmania* species, each exhibiting distinct drug susceptibility patterns, further complicates the development of a universal treatment strategy [9,10]. The quest to identify novel treatment modalities for CL has been hindered by the intricate parasite–host interactions, variable immune responses and diverse *Leishmania* species that underpin this disease. Consequently, research endeavors have been directed towards the identification of compounds exhibiting potent anti-leishmanial activity, both in vitro and in vivo, to address the limitations of current therapeutic interventions [10,11].

*N*-Heterocyclic carbenes (NHCs) are widely recognized as versatile ligands in both organometallic and inorganic chemistry, primarily due to their strong σ-donating properties. These properties result in more stable metal-ligand bonds. The lone pair of electrons on the carbene carbon is stabilized by the two adjacent nitrogen atoms through inductive effects [12,13]. NHCs are compounds of interest for study due to their enhanced stability in the presence of moisture and air, as well as their robust coordination with transition metals [14]. In addition, it has been demonstrated that alterations in the nitrogen atom substituents of the NHC ligands can result in a substantial change in their reactivity and binding strength with metal centers [15]. Extensive research has been conducted on metal-NHC complexes based on gold, copper and especially silver due to their applications in catalysis, materials science and medicinal chemistry [16,17,18,19,20,21,22,23].

Recent investigations have underscored the auspicious antimicrobial properties of silver-NHC complexes [24,25,26]. These findings are consistent with a growing body of literature demonstrating that silver(I) complexes stabilized by *N*-heterocyclic carbene ligands also exhibit promising antiparasitic activity against various protozoan pathogens. There is accumulating evidence that Ag(I)-NHC complexes are active against *Leishmania* species, *Trypanosoma cruzi* and *Acanthamoeba castellanii*. For instance, Ag(I)-NHC complexes with hybrid sulfonamide/thiourea ligands have been reported to exhibit enhanced leishmanicidal activity against *L. infantum* and *L. braziliensis*, demonstrating favorable selectivity profiles [27]. Similarly, in vitro evaluation of selected Ag(I)-NHC architectures against *L. major* promastigotes revealed clear, concentration-dependent inhibition of parasite viability [28]. Beyond leishmaniasis, Ag(I)-NHC complexes have demonstrated potent antiparasitic activity against *T. cruzi*, which is potentially mediated by the disruption of parasite redox homeostasis through the inhibition of trypanothione reductase [29]. Furthermore, benzimidazole-based Ag(I)-NHC derivatives have been reported to exhibit significant trophocidal and cysticidal activities against clinical isolates of *A. castellanii* [30]. Taken together, these studies emphasize the increasing potential of silver-NHC complexes as promising candidates for developing new therapeutic strategies against neglected tropical diseases.

**Figure 1:**
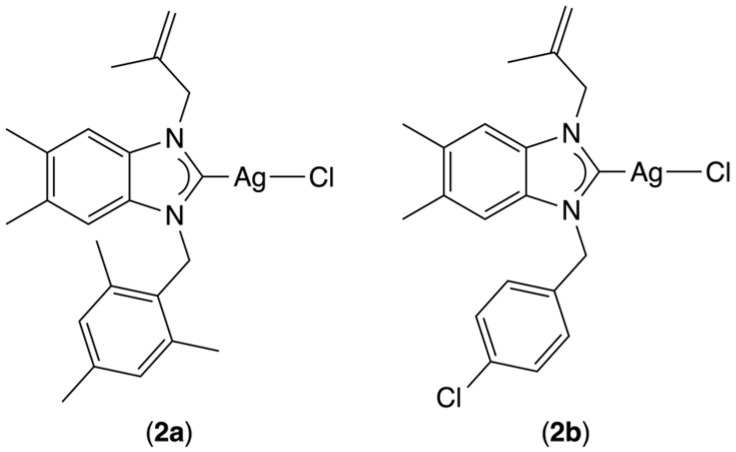
Silver(I) complexes **2a**,**2b** studied against *Leishmania major*.

In the present study, a thorough assessment of the therapeutic potential of two silver-NHC complexes, namely chloro[1-methallyl-3-(2,4,6-trimethylbenzyl)-5,6-dimethylbenzimidazole-2-ylidene]silver(I) (**2a**) and chloro[1-methallyl-3-(4-chlorobenzyl)-5,6-dimethylbenzimidazole-2-ylidene]silver(I) (**2b**) [31] (Figure 1), was conducted. This evaluation was based on in vitro and in vivo studies on *Leishmania* major promastigotes using an experimental model of CL in Balb/c mice. The objective of these integrations was twofold: first, to provide solutions to the limitations of existing treatments and second, to contribute to the ongoing search for safer and more effective therapeutic strategies for CL. Furthermore, an elaborate investigation was conducted into the two silver(I) complexes **2a**,**2b** and their benzimidazolium salt precursors **1a**,**1b**, employing the molecular docking method. This computational research was conducted using a set of proteins and enzymes, including LaGP63, LaARG, *N*-myristoyltransferase and farnesyl pyrophosphate synthase. It is widely accepted that the molecular docking method provides a solid theoretical framework for the design of new molecular scaffolds. These in silico analyses were also used to make predictions, which then guided the synthesis of new enzyme inhibitors with potential activity [32,33].

## 2. Results and Discussion

### 2.1. Synthesis and Characterization of Silver(I) Complex ***2a***

**Scheme 1:**
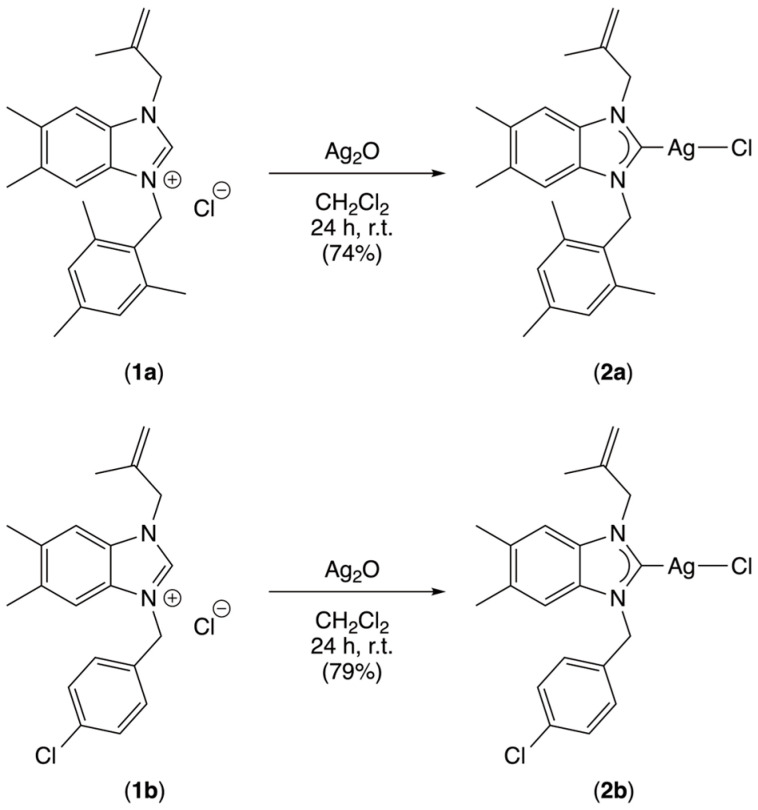
Synthesis of silver(I) complexes **2a** and **2b**.

The chloro[1-methallyl-3-(2,4,6-trimethylbenzyl)-5,6-dimethylbenzimidazole-2-ylidene]silver(I) (**2a**) and chloro[1-methallyl-3-(4-chlorobenzyl)-5,6-dimethylbenzimidazole-2-ylidene]silver(I) (**2b**) complexes were obtained by stoichiometric reaction between 1-methallyl-3-(2,4,6-trimethylbenzyl)-5,6-dimethylbenzimidazolium chloride (**1a**) or 1-methallyl-3-(4-chlorobenzyl)-5,6-dimethylbenzimidazolium chloride (**1b**), respectively, and silver oxide (Ag_2_O) in dichloromethane, with the reaction being protected from exposure to light. Following a 24-h period of ambient temperature, the solutions were filtered and subsequently concentrated under vacuum. The complexes **2a** and **2b** were isolated in 74 and 79% yield, respectively, by precipitation with diethyl ether (Scheme 1).

The silver(I) complexes **2a** and **2b** were soluble in common organic solvents, such as *N*,*N*-dimethylformamide, dichloromethane, chloroform, ethanol and acetonitrile. However, the complexes were insoluble in hydrocarbons and ethers. The structural characteristics of the complexes were subsequently elucidated through nuclear magnetic resonance (NMR) spectroscopy. Their ^1^H NMR spectra confirmed the assigned structures. The resonance for characteristic NCHN protons of the benzimidazolium salts **1a** and **1b**, observed as sharp singlets at 11.25 and 11.82 ppm, respectively, was not detected. Furthermore, the methallyl ligand was characterized by the presence of four singlets at approximately 1.7, 4.7, 4.9 and 4.9 ppm, accompanied by relative intensities of 3, 1, 2 and 1, respectively. In contrast, the 2,4,6-trimethylbenzyl and 4-chlorobenzyl substituents exhibited a singlet at 5.41 and 5.54 ppm, respectively, attributable to their CH_2_. The trimethyl substituents of the aromatic ring were characterized by singlets at 2.23 and 2.33 ppm with relative intensities of 6 and 3, respectively. ^13^C{^1^H} NMR spectra analysis revealed the absence of the NC(Ag)N peaks, likely due to the fluxional nature of the silver complex [34,35,36]. However, signals attributable to the substituents of the dimethylbenzimidazolin-2-ylidene were discernible at 20.09, 56.05, 111.85 and 139.64 ppm for the methallyl moiety and 20.45 and 20.51 for the CH_3_ of the mesityl function for complex **2a**. In the case of complex **2b**, the signals from the methallyl substituent were discernible at 20.18, 55.50, 112.10 and 139.29 ppm.

In addition, FT-IR analysis of the corresponding benzimidazolium salts **1a** and **1b** revealed sharp CN stretching bands at 1549 [37] and 1558 cm^−1^ [31], respectively. Conversely, the corresponding signals for the silver(I) complexes **2a** and **2b** exhibited a shift to lower wavenumbers at 1393 and 1388 cm^−1^, respectively. In addition, FT-IR analysis of the corresponding benzimidazolium salt **1a** revealed sharp CN stretching bands at 1549 cm^−1^ [37]. Conversely, the corresponding signals for the silver(I) complex **2a** exhibited a shift to lower wavenumbers at 1393 cm^−1^. This shift is indicative of the weakening of the carbon-nitrogen bond, which underwent a partial double bond after coordination in agreement with literature values [31,38]. The Supplementary Information section contains the FT-IR, ^1^H and ^13^C{^1^H} NMR spectra (Figures S1–S6).

The two silver(I) complexes **2a**,**2b** were sensitive to light but stable to air and moisture in the solid state. The stability of the complex **2b** in solution (DMSO-*d*_6_) was evaluated by recording its ^1^H NMR spectra at regular intervals over a period of five days. During this period, the sample was stored in darkness to prevent light-induced alterations. The NMR spectra exhibited no variations in either the chemical shifts or the signal intensities throughout the monitoring period, indicating that the complexes remained stable in solution (Supplementary Materials, Figure S7).

### 2.2. Cytotoxic Potential of Silver(I) Complexes

**Figure 2:**
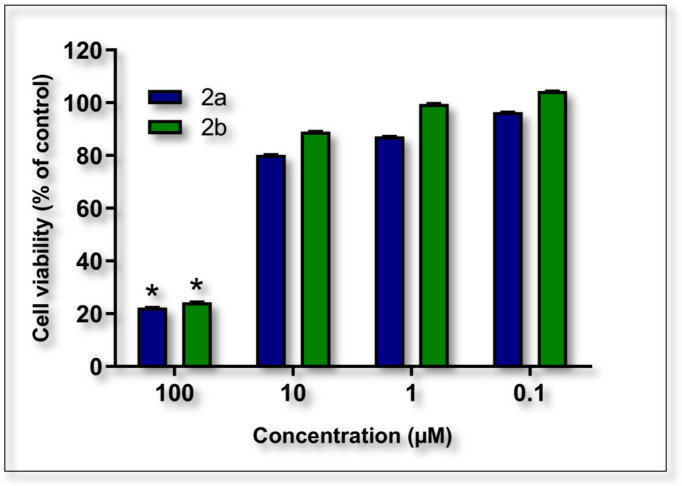
Cytotoxicity of 10^−4^, 10^−5^, 10^−6^ and 10^−7^ M concentrations of silver complexes **2a**,**2b** against the L929 mouse fibroblast cell line in vitro using the XTT method. * *p* < 0.05 vs. control.

The assessment of cellular toxicity was conducted on L929 cells following a 24-h exposure to silver(I) complexes **2a**,**2b**. No significant reduction in cell viability was observed at concentrations of 10^−5^, 10^−6^ and 10^−7^ M compared to untreated controls (*p* > 0.05). However, treatment at 10^−4^ M resulted in a significant decrease in cell viability (*p* < 0.05), indicating concentration-dependent toxicity (Figure 2). Subsequent anti-leishmanial assays were conducted at concentrations of 10^−5^, 10^−6^ and 10^−7^ M, which ensured the maintenance of a non-toxic profile in mammalian cells. L929 cells exhibited viability of 80 and 22% at 10^−5^ and 10^−4^ M, respectively, for complex **2a** and 85 and 25% at 10^−5^ and 10^−4^ M, respectively, for complex **2b**. In light of the findings, the CC_50_ values were ascertained to be approximately 33 µM and 40 µM for silver(I) complexes **2a** and **2b**, respectively, by means of log-linear interpolation. The solvent control (2% dimethyl sulfoxide) exerted no statistically significant effect on the viability of L929 fibroblasts in comparison with the untreated control (*p* > 0.05). Consequently, the decline in cell viability observed at elevated concentrations of the tested compounds was attributable to the inorganic compounds themselves rather than the solvent.

### 2.3. In Vitro Effects of L. major Promastigotes

**Figure 3:**
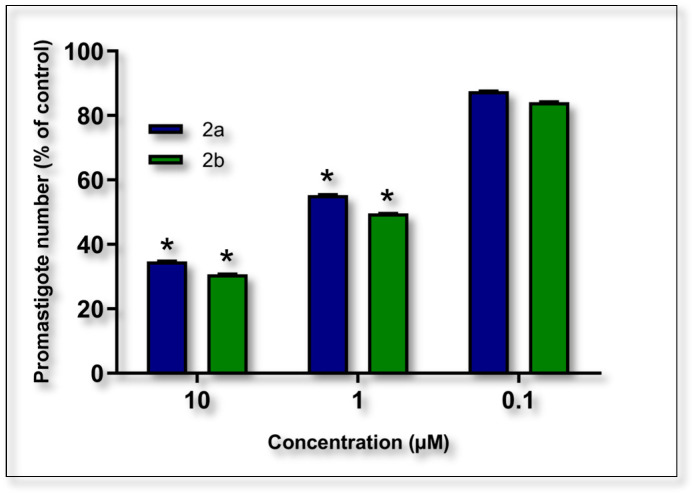
In vitro activity of 10^−5^, 10^−6^ and 10^−7^ M concentrations of silver complexes **2a**,**2b** against *L. major* promastigotes.* *p* < 0.05 vs. control.

In comparison with the control group, silver(I) complexes **2a**,**2b** demonstrated a dose-dependent inhibitory effect on the proliferation of *L. major* promastigotes. A substantial inhibition was observed in both complexes at concentrations of 10^−5^ and 10^−6^ M (*p* < 0.01; Figure 3). The results of the dose–response analysis indicated that the IC_50_ values for complexes **2a** and **2b** were 12 µM and 17 µM, respectively. This finding suggests that complex **2a** is slightly more potent than complex **2b**. The Selectivity Index, calculated from the CC_50_ values on L929 fibroblasts, was approximately 2.8 for complex **2a** and 2.4 for complex **2b**. This finding indicates that both inorganic compounds demonstrate anti-leishmanial activity at concentrations that are below their respective cytotoxic thresholds. The 2% dimethyl sulfoxide solvent control exhibited no statistically significant effect in comparison with the untreated control (*p* > 0.05), thereby confirming that the observed inhibition was attributable to the test silver(I) complexes. Note that the anti-*leishmania* activity of the most effective complex (complex **2b**) exhibited slightly higher efficacy compared with that reported for the chloro[1-methallyl-3-(2,3,5,6-tetramethylbenzyl)¡5,6-dimethylbenzimidazole-2-ylidene]silver(I) complex [39].

A previous study of ours demonstrated that NHC precursors alone exhibited minimal amoebicidal activity, suggesting that the observed antiamoebicidal effects are attributable to the addition of the silver atom. This finding underscores the pivotal function of silver coordination in modulating activity against pathogenic *Acanthamoeba trophozoites* [40]. Ashraf and co-workers conducted a study to assess the impact of the silver center on anticancer activity. They compared the biological properties of the corresponding benzimidazolium salts with those of their Ag-NHC complexes. The findings indicated that the silver(I) complexes exhibited notably higher anticancer activity in comparison to the benzimidazolium salts [41]. A study conducted in 2020 reported that silver-NHC complexes exhibited higher activity compared to benzimidazolium salts [42]. In light of the numerous biological studies conducted, it can be concluded that silver metal exerts a significant effect on biological activity in the study presented herein.

### 2.4. In Vivo Anti-Leishmanial Activity

In mice infected with *L. major*, the inflammation of the plantar regions of the feet commenced at the termination of the first week and attained its maximum extent during the third week post-infection. The dimensions of the lesions (length and width) were measured on a weekly basis and the areas of the lesions were calculated. At the initiation of treatment, the mean lesion size was 52.3 ± 9.1 mm^2^, with comparable baseline values observed across all groups (Table 1).

**Figure 4:**
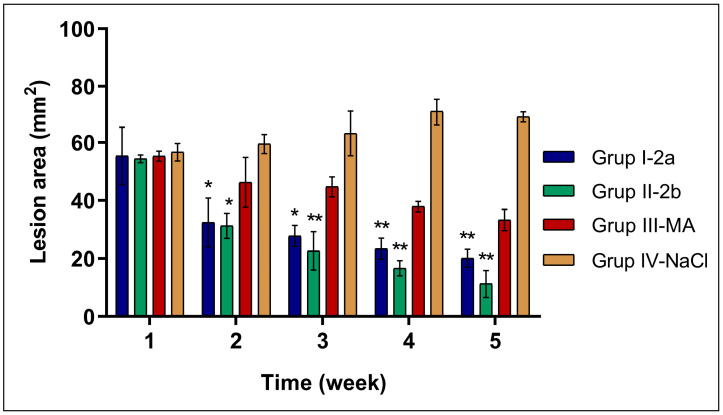
Mean footpad lesion size (mm^2^) of groups I–IV. Data were described using median, minimum and maximum. * *p* < 0.05 vs. Group III-MA; ** *p* < 0.01 vs. Group III-MA.

**Figure 5:**
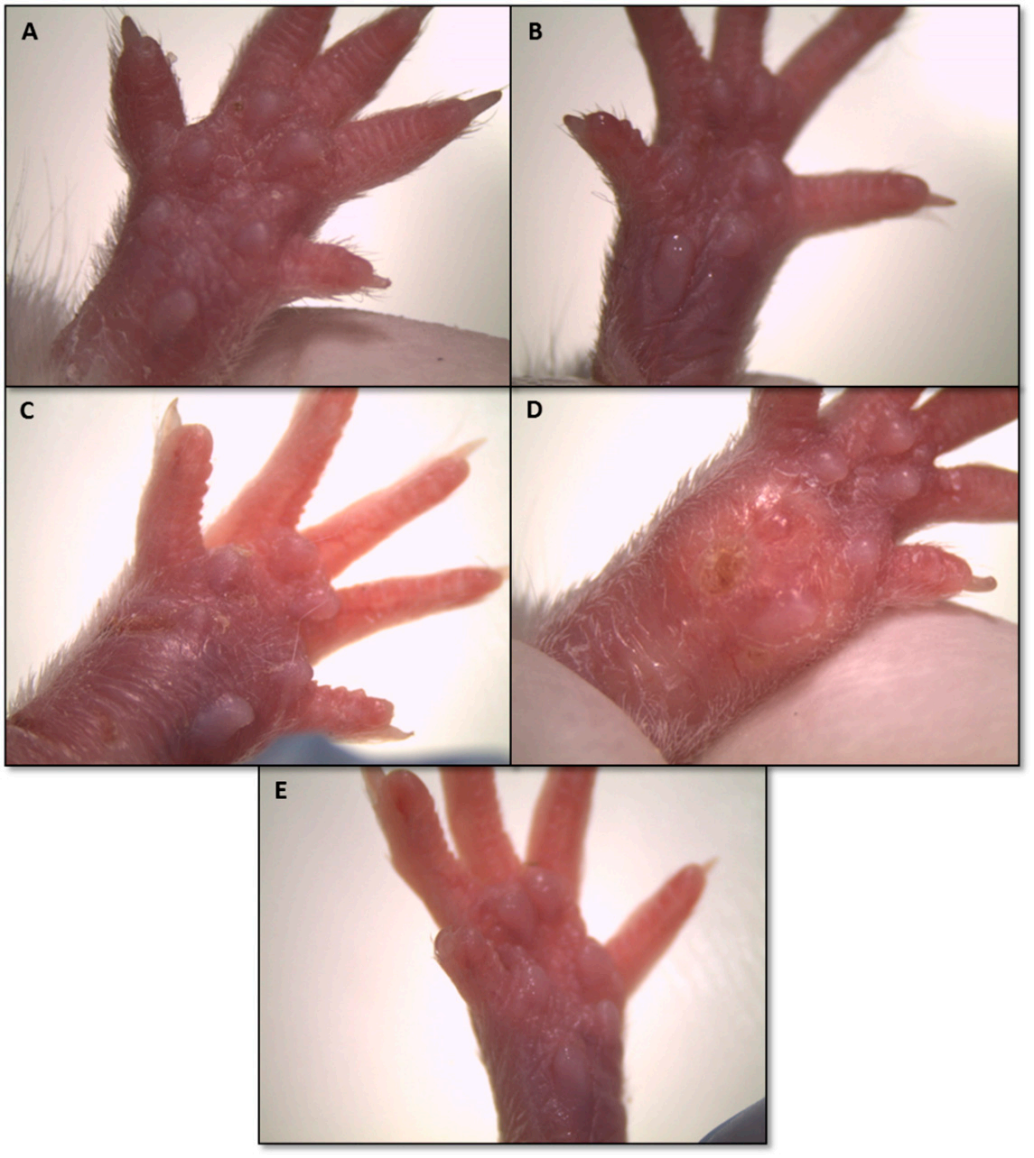
Appearance of silver(I) complexes **2a** (**A**), **2b** (**B**), positive control (**C**) and negative control (**D**) lesions on the footpads of Balb/c mice at week 4 of treatment in an experimentally induced CL model. Normal appearance of Balb/c footpads, non-infected control group (**E**).

Treatment with silver(I) complexes **2a** (group I) and **2b** (group II) led to a substantial reduction in lesion size when compared with the untreated infected group. The therapeutic response was evaluated in relation to the standard treatment for CL, meglumine antimoniate (MA). The present study demonstrated that MA significantly diminished the size of lesions and the parasite burden, thereby establishing itself as a clinically relevant benchmark. A comparison of the experimental complexes with the MA group (group III) revealed that both produced significantly different lesion sizes. By week 5, the mean lesion size was 20.9 ± 3.1 mm^2^ in group I (treated with complex **2a**), 11.2 ± 4.6 mm^2^ in group II (treated with complex **2a**), 33.2 ± 3.6 mm^2^ in group III (treated with MA) and 68.8 ± 1.7 mm^2^ in the untreated control group (Table 2; Figure 4 and Figure 5).

In this study, MA was administered at a dose of 20 mg/kg/day, representing the most commonly used and clinically relevant reference regimen in experimental models of CL [5]. Although higher doses of antimony have been reported in select preclinical studies, such regimens are often associated with increased systemic toxicity, including cardiotoxicity and nephrotoxicity [9]. From a translational perspective, the development of alternative anti-leishmanial agents that can achieve therapeutic efficacy at lower or comparable doses remains a clinical priority. While the present data demonstrate the in vivo antileishmanial activity of the synthesized silver(I)-NHC complexes using a standard reference dose, future dose–response studies will incorporate higher benchmark doses of MA (e.g., 50–100 mg/kg) to further define the comparative therapeutic window of these candidates.

During the in vivo treatment period, the mice were closely monitored for any adverse effects, including alterations in body weight, behavior and local reactions at the injection site. The administration of the silver(I)-NHC complexes at the prescribed dosage did not result in any discernible indications of systemic toxicity, treatment-related mortality or severe local adverse reactions in the animal subjects. While these observations suggest a favorable preliminary in vivo tolerability profile, comprehensive toxicological evaluations, including histopathological and biochemical analyses, will be required in future studies to fully characterize the safety profile of these compounds.

The selectivity indices (SI) obtained from in vitro assays were relatively modest (approximately 2–3) in comparison with those of some previously reported antileishmanial compounds. However, despite the moderate in vitro values, the silver(I)-NHC complexes **2a**,**2b** demonstrated clear therapeutic efficacy in vivo. This finding suggests that additional pharmacodynamic or host-related factors may contribute to their antileishmanial activity.

The present study focused on evaluating therapeutic efficacy during the active treatment period and shortly afterwards. Although the size of the lesions was monitored for up to one week after treatment was completed, longer-term follow-up could provide valuable information about relapse, the durability of the response, and long-term disease control. Consequently, subsequent studies will encompass extended monitoring periods following treatment cessation to more accurately assess sustained therapeutic outcomes.

### 2.5. Histopathological Evaluation

**Figure 6:**
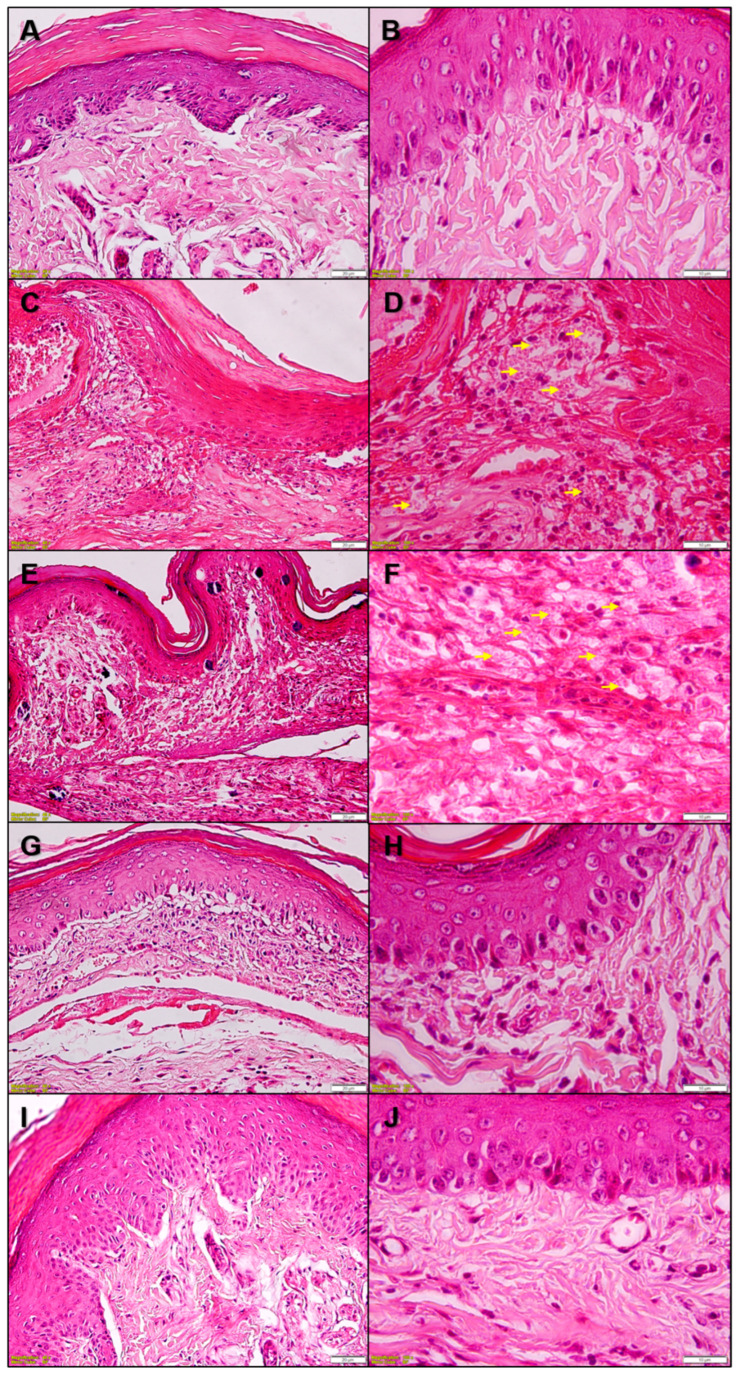
Representative photomicrographs of histological sections of foot tissue from BALB/c mice across the study groups, stained with haematoxylin and eosin (H&E). (**A**,**B**) non-infected control; (**C**,**D**) negative control; (**E**,**F**) positive control; (**G**,**H**) **2a**-treated; (**I**,**J**) **2b**-treated. Images were captured at ×40 magnification (**A**,**C**,**E**,**G**,**I**) and ×100 magnification (**B**,**D**,**F**,**H**,**J**). *Leishmania amastigotes* within cells are indicated by yellow arrows.

Histopathological analysis of paw tissues from the non-infected control group revealed intact epithelial and basement membrane integrity with well-organized collagen fibers (Figure 6A,B). Conversely, the infected, untreated group exhibited disrupted epithelial formation, dense polymorphonuclear leukocyte infiltration indicative of inflammation, the presence of abundant amastigotes and disorganized collagen fibers (Figure 6C,D).

In the positive control group, which received MA treatment, there was a notable preservation of tissue integrity and collagen formation. A comparative analysis of the experimental group and the negative control group revealed a lower density of amastigotes, despite the persistence of pronounced inflammatory infiltration (Figure 6E,F).

Treatment with silver(I) complexes **2a** (Figure 6G,H) and **2b** (Figure 6I,J) resulted in the maintenance of tissue integrity and collagen architecture, with no detectable amastigotes and only minimal inflammatory changes. A comparative assessment indicated more uniform tissue preservation in the group treated with complex **2b**. Despite the in vitro assays being conducted under neutral pH conditions, the therapeutic effects observed in vivo suggest that the silver complexes may be active within the acidic phagolysosomal environment of macrophages, where amastigotes persist. Further investigation is warranted to clarify the stability and mechanism of action of the complexes under acidic intracellular conditions.

### 2.6. Molecular Docking Analysis

The treatment of *leishmaniasis* continues to necessitate the identification of novel and effective drug targets. Enzymes present in the parasite but absent from their mammalian host are considered ideal targets for rational drug design. Leishmanolysin, also termed LaGP63, is a glycolprotein that contributes to parasite virulence and pathogenesis [43]. Consequently, GP63 has been identified as both a drug target and a vaccine candidate. Another essential enzyme in the polyamine biosynthetic pathway is arginase (LaARG). This enzyme is imperative for parasite survival, as it plays a pivotal role in regulating reactive oxygen species-induced apoptosis [44]. Consequently, the inhibition of LaARG results in mitochondrial dysfunction, which ultimately leads to parasite death. *N*-Myristoyltransferase, an enzyme, has been shown to play a role in parasitic metabolic processes of *L. major*. In 1997, it was determined that *N*-myristoyltransferase is essential for the survival of *Leishmania* sp. and the importance of this enzyme has been investigated in various organisms [45]. A multitude of studies have demonstrated the efficacy of molecules such as pyrrolidines, piperidinyldinodols, azetidinopyrimidines, aminomethylindazoles, benzimidazoles, and thienopyrimidines in inhibiting *N*-myristoyltransferase. Consequently, there has been a surge in research endeavors focused on the development of novel molecules derived from these structural frameworks [46]. One of the strategies for the control of parasitic infections is the sterol biosynthesis pathway. Numerous studies on this pathway have yielded remarkable results against various trypanosomatids, including *L. major*. Farnesyl pyrophosphate synthase is one of the necessary enzymes for isoprenoid synthesis. The inhibition of this enzyme disrupts the lipid layer of cells, leading to the death of parasites [47]. Consequently, farnesyl pyrophosphate synthase emerges as a pivotal target molecule for anti-leishmanial studies.

In the present case, the interactions of silver(I) complexes **2a**,**2b** and their benzimidazolium salts, 1-methallyl-3-(2,4,6-trimethylbenzyl)-5,6-dimethylbenzimidazolium chloride (**1a**) and 1-methallyl-3-(4-chlorobenzyl)-5,6-dimethylbenzimidazolium chloride, were in silico analyzed against LaGP63, LaARG, *N*-myristoyltransferase and farnesyl diphosphate synthase using the molecular docking method (Table 2).

According to the results obtained against LaGP63, the most effective inhibitor should be complex **2a**, exhibiting a binding affinity of −6.98 kcal/mol. The silver(I) complexes **2a**,**2b** exhibited higher binding affinities in comparison to their corresponding benzimidazolium salts **1a**,**1b**. It has been calculated that benzimidazolium salt **1b** (−6.23 kcal/mol) exhibited a stronger binding affinity than salt **1a** (−5.40 kcal/mol) (Table 2).

**Figure 7:**
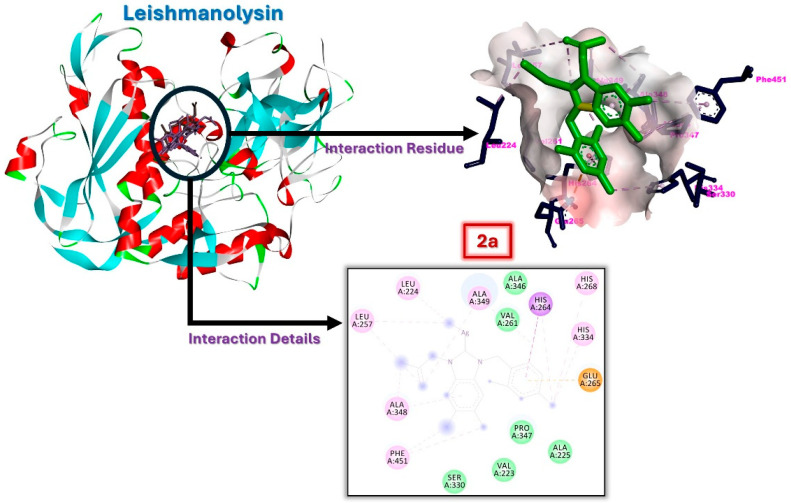
Interaction details and residues of complex **2a** against LaGP63.

Ansari and co-workers conducted a thorough analysis of benzimidazole derivative molecules, focusing on their interaction with the LaGP63 structure. In this case, the utilization of the site finder option of the Molecular Operating Environment program is indicated, given the absence of a co-crystallized ligand. A comprehensive record of interactions with the residue was obtained, encompassing multiple interactions with His264, Asp342, His268, His334, Met345, Ala348, Phe272 and Ser333 [48]. The benzimidazolium salts **1a**,**1b** and silver(I) complexes **2a**,**2b** exhibited interaction with the same residue of the target molecule. This finding aligned with the results reported by Ansari. Complex **2a** displayed π-interactions with His264 and Glu265, in addition to alkylic interactions with His224, Leu268, His334, Ala348, Ala349 and Phe451 and numerous van der Waals interactions (Figure 7). The findings of this study demonstrated congruence with those of other research endeavors in this field.

In particular, Mercado-Camargo, Vivas-Reyes and co-workers analyzed the orientation between biflavonoids and LaGP63 and determined remarkable interactions with His264, His268, His334, Met345, Ala348, Ala227, Ser273, Gly222, Thr228, Glu265, Ala350, Pro347, Val223, Gly329 and Glu220 [49]. In addition, Bispo and co-workers confirmed the anti-leishmanial activity of *N*^1^,*N*^2^-disubstituted benzoylguanidines using molecular docking methods against LaGP63. They recorded interactions with the residue, including His263, Glu264, Ala328, Pro344 and Ala346 amino acids of LaGP63. Additionally, an analysis was conducted on the tautomeric molecules in relation to LaARG, leading to the determination of interactions with His114, Asp141, Asn143, His154 and Asp194 (vide infra) [50]. The interaction types and binding values with salts **1a**,**1b** and complexes **2a**,**2b** were presented in Table 2 and the details of all interactions were also presented in Figures S8–S11 (Supplementary Materials).

**Figure 8:**
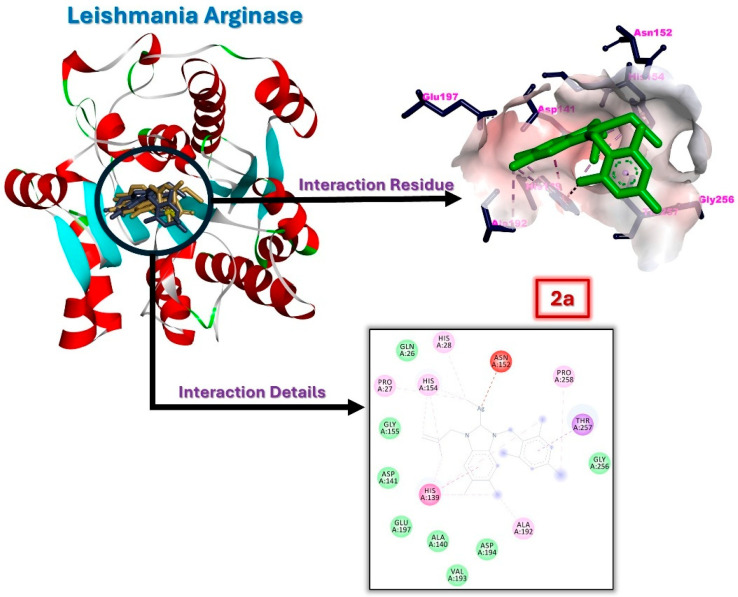
Interaction details and residues of complex **2a** against LaARG.

An evaluation of the interactions between benzimidazolium salts **1a**,**1b** and their silver(I) complexes **2a**,**2b** with LaARG revealed that complex **2a** displayed the optimal binding affinity of −4.47 kcal/mol, exhibiting π-interactions with His139 and Thr257 and alkylic interactions with Pro27, His28, His154, Ala192 and Pro258, in addition to numerous van der Waals interactions (Figure 8). The results of the simulations conducted against LaARG indicate that the silver complexes **2a**,**2b** should exhibit superior activity in comparison to the benzimidazolium salts **1a**,**1b**. It was observed that all of the four molecules interacted with the same part of the target protein (Table 2). The details of the interactions were presented in Figures S12–S15 (Supplementary Materials).

**Figure 9:**
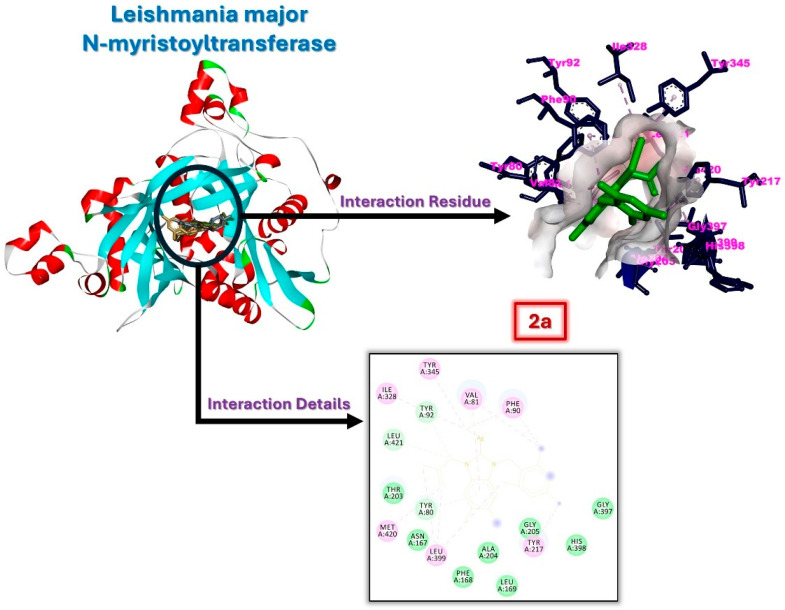
Interaction details and residues of complex **2a** against *N*-myristoyltransferase.

*N*-Myristoyltransferase, an enzyme implicated in the metabolic processes of *L. major*, has been identified as being essential for parasite survival. Its critical role in *Leishmania* species has been the subject of extensive study in a variety of organisms. In their analysis, Tate and co-workers examined the potential of thienopyrimidine to inhibit *N*-myristoyltransferase [51]. More recently, Orabi and co-workers analyzed the 10 alkaloids, 15 phenolic compounds, 10 sterols, 6 withanones, 6 chlorinated withanolides, 5 sulfur-containing withanolides, 9 withanamides, 87 withanolides, and 19 withanosides present in *Withania somnifera* to identify a possible *N*-myristoyltransferase inhibitor. They recorded the highest affinity for calycopteretin-3-rutinoside and withanoside IX, with interactions through Tyr92, Phe90, Asn167, Phe88, Thr203, His219, Tyr217, Phe232, Tyr326, Val81, Tyr80, Ser330, Leu341, Asp396 and Asn376 [52]. The benzimidazolium salts **1a**,**1b** and their silver(I) complexes **2a**,**2b** interacted with the cited above residue of the target *N*-myristoyltransferase. The interaction area was confirmed by the co-crystalized ligand, thienopyrimidine, with a root mean square deviation (RMSD) of ≤2 Å. The stronger binding affinity was determined for complex **2a** with a binding affinity of −8.27 kcal/mol with H-bonds, alkylic and van der Waals interactions. The H-bonds of complex **2a** were recorded with Tyr80, Tyr92 and Leu421 (Figure 9), while complex **2b** has only one H-bond with Thr203. The results of the study indicated that silver(I) complexes **2a**,**2b** exhibited stronger binding values compared to those of the corresponding salts **1a**,**1b** (Table 2 and Figures S16–S19 in Supplementary Materials).

Farnesyl pyrophosphate synthase is a pivotal enzyme in isoprenoid biosynthesis. Its inhibition has been shown to disrupt parasite cell membranes, leading to parasite death. This makes farnesyl pyrophosphate synthase an important anti-leishmanial drug target. Dos Santos Junior and co-workers sought to identify potential inhibitors for farnesyl diphosphate synthase using in silico methods, such as pharmacophore modeling and molecular docking. It was determined that effective inhibitors interacted with the residue, including Lys207, Lys264, Arg107, Asp102, Asp98, Asp99, Asp250, Phe94, Leu95, His93, Met101 and Gln167 [53]. In their analysis of nitrogen-containing bisphosphonate molecules with farnesyl diphosphate synthase, Semenyuta and co-workers observed interactions occurring with Asp99, Asp170, Lys196, Asp257 and Lys262 residues [54].

**Figure 10:**
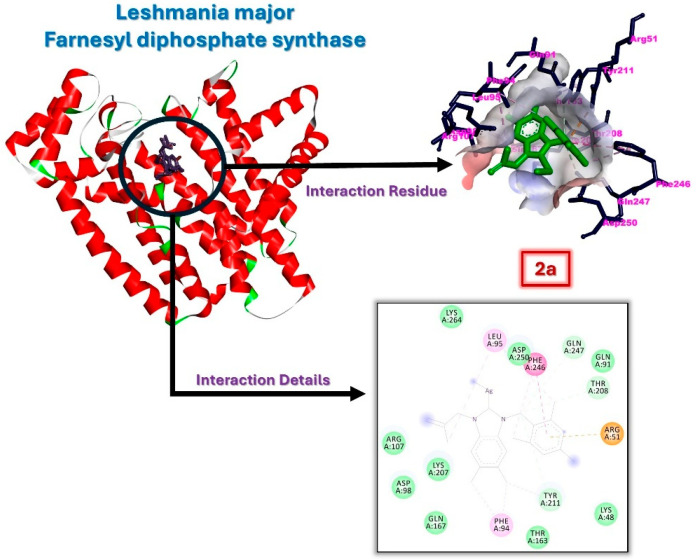
Interaction details and residues of complex **2a** against farnesyl diphosphate synthase.

The four molecules of interest **1a**,**1b** and **2a**,**2b** interacted with the same sets of residues. The interaction area was confirmed by the co-crystalized ligand, 3-fluoro-1-(2-hydroxy-2,2-diphosphonoethyl)pyridinium, with ≤2 Å RMSD. The stronger binding affinity was determined for complex **2a**, with a value of −5.35 kcal/mol, involving H-bonds, alkylic and van der Waals interactions (Figure 10). The H-bonds of complex **2a** were recorded with Thr208, Tyr211 and Gln24, while complex **2b** has H-bonds with the same amino acids (Table 2). The findings of the study demonstrated that, once again, silver(I) complexes **2a**,**2b** exhibited superior binding values in comparison to those of the corresponding salts **1a**,**1b**. The details of the interactions were presented in Figures S20–S23 (Supplementary Materials).

## 3. Materials and Methods

### 3.1. Chemistry

#### 3.1.1. General

The reagents utilized in the procedures were obtained commercially from Sigma-Aldrich. The melting points were measured in air using an Electrothermal 9200 (Electrothermal Engineering Ltd., Essex, UK) apparatus equipped with glass capillaries; the reported value was not corrected. Fourier transform infrared (FT-IR) spectrum was recorded on a Perkin Elmer 100 spectrometer (Waltham, MA, USA). Nuclear magnetic resonance (NMR) spectra were obtained using a Bruker Avance III 400 MHz spectrometer (Bruker Corporation, Billerica, MA, USA). The calibration of spectra was performed in accordance with the residual protonated solvent for CDCl_3_, with a proton resonance frequency of 7.26 ppm and 77.16 ppm for ^1^H and ^13^C{^1^H}, respectively. The chemical shifts and coupling constants are expressed in ppm and Hz, respectively. 1-Methallyl-3-(2,4,6-trimethylbenzyl)-5,6-dimethylbenzimidazolium chloride (**1a**) [31], 1-methallyl-3-(4-chlorobenzyl)-5,6-dimethylbenzimidazolium chloride (**1b**) [26] and chloro[1-methallyl-3-(4-chlorobenzyl)-5,6-dimethylbenzimidazole-2-ylidene]silver(I) (**2b**) [26] were synthesized in accordance with our previous work.

#### 3.1.2. Preparation of Silver Complexes **2a**,**2b**

In a Schlenk flask, an aluminum foil-covered tube under argon, silver(I) oxide (Ag_2_O, 2.2 mmol) was added to a stirring solution of benzimidazolium salt (1.0 mmol) in dichloromethane (50 mL). Following a 24-h period at room temperature, the mixture underwent filtration. The clear solution was concentrated by vacuum pressure until approximately 5 mL, at which point the silver complex was precipitated by the addition of diethyl ether (50 mL). Following a filtration process and subsequent vacuum drying, the silver complex was isolated as a white solid.

Chloro[1-methallyl-3-(2,4,6-trimethylbenzyl)-5,6-dimethylbenzimidazole-2-ylidene]silver(I) (**2a**): yield 74%; m.p. 247–248 °C, FT-IR: ν(CN): 1393 cm^−1^. ^1^H NMR (400 MHz, CDCl_3_): *δ* = 1.67 (s, 3H, NCH_2_C(C*H*_3_)=C*H*_2_), 2.22 (s, 6H, C_6_H_2_(C*H*_3_)_3_), 2.33 (s, 3H, C_6_H_2_(C*H*_3_)_3_), 2.34 (s, 3H, (C*H*_3_)_2_-C_7_H_2_N_2_), 2.35 (s, 3H, (C*H*_3_)_2_-C_7_H_2_N_2_), 4.73 (s, 1H, NCH_2_C(C*H*_3_)=C*H*_2_), 4.86 (s, 2H, NC*H*_2_C(C*H*_3_)=CH_2_), 4.95 (s, 1H, NCH_2_C(C*H*_3_)=C*H*_2_), 5.41 (s, 2H, NC*H*_2_C_6_H_2_(C*H*_3_)_3_), 6.97 (s, 2H, arom CH of C_6_*H*_2_(C*H*_3_)_3_), 7.07 (s, 1H, arom CH of (C*H*_3_)_2_-C_7_*H*_2_N_2._), 7.16 (s, 1H, arom CH of (C*H*_3_)_2_-C_7_*H*_2_N_2._); ^13^C{^1^H} NMR (100 MHz, CDCl_3_): *δ* (ppm): 20.10 (s, NCH_2_C(C*H*_3_)=C*H*_2_), 20.45 (s, C_6_H_2_(C*H*_3_)_3_), 20.51 (s, C_6_H_2_(C*H*_3_)_3_), 20.62 (s, C*H*_3_)_2_-C_7_H_2_N_2_), 21.29 (s, C*H*_3_)_2_-C_7_H_2_N_2_), 47.51 (s, N*C*H_2_C_6_H_2_(C*H*_3_)_3_), 56.05 (s, N*C*H_2_C(C*H*_3_)=C*H*_2_), 111.85 (s, NCH_2_C(C*H*_3_)=C*H*_2_), 112.22, 114.11, 126.88, 130.26, 132.62, 133.01, 133.61, 133.87, 137.55, 139.42 (10s, arom Cs), 139.64 (s, NCH_2_*C*(C*H*_3_)=C*H*_2_) ppm. Anal. Calcd. for C_23_H_28_ClN_2_Ag: C, 58.06; H, 5.93; N, 5.89. Found C: 57.92; H: 6.16; N: 5.91.

Chloro[1-methallyl-3-(4-chlorobenzyl)-5,6-dimethylbenzimidazole-2-ylidene]silver(I) (**2b**): yield 79%; m.p. 105–106 °C, FT-IR: ν(CN): 1388 cm^−1^. ^1^H NMR (400 MHz, CDCl_3_): *δ* = 1.74 (s, 3H, NCH_2_C(C*H*_3_)=C*H*_2_), 2.30 (s, 3H, (C*H*_3_)_2_-C_7_H_2_N_2_), 2.34 (s, 3H, (C*H*_3_)_2_-C_7_H_2_N_2_), 4.79 (s, 1H, NCH_2_C(C*H*_3_)=C*H*_2_), 4.92 (s, 2H, NC*H*_2_C(C*H*_3_)=C*H*_2_), 5.02 (s, 1H, NCH_2_C(C*H*_3_)=C*H*_2_), 5.54 (s, 2H, NC*H*_2_C_6_H_4_Cl), 7.03 (s, 1H, arom CH of (C*H*_3_)_2_-C_7_*H*_2_N_2._), 7.15 (s, 1H, arom CH of (C*H*_3_)_2_-C_7_*H*_2_N_2._), 7.18 (d, 2H, arom CH of C_6_H_4_Cl, ^3^*J*_HH_ = 8.4 Hz), 7.29 (d, 2H, arom CH of C_6_H_4_Cl, ^3^*J*_HH_ = 8.4 Hz); ^13^C{^1^H} NMR (100 MHz, CDCl_3_): *δ* = 20.18 (s, NCH_2_C(C*H*_3_)=C*H*_2_), 20.54 (s, C*H*_3_)_2_-C_7_H_2_N_2_), 20.55 (s, C*H*_3_)_2_-C_7_H_2_N_2_), 52.70 (s, N*C*H_2_C_6_H_4_Cl), 55.50 (s, N*C*H_2_C(C*H*_3_)=C*H*_2_), 112.10 (s, NCH_2_C(C*H*_3_)=C*H*_2_), 112.38, 114.49, 128.42, 129.40, 132.18, 132.82, 133.80, 134.13, 134.46 (9s, arom Cs), 139.29 (s, NCH_2_*C*(C*H*_3_)=C*H*_2_) ppm. Anal. Calcd. for C_20_H_21_Cl_2_N_2_Ag: C, 51.31; H, 4.52; N, 5.98. Found C: 51.15; H: 4.37; N: 5.78.

### 3.2. Cell Culture and Cytotoxicity Evaluation

#### 3.2.1. Cell Line and Culture

The mouse fibroblast cell line (L929, ATCC, NCTC clone 929) was identified as the optimal cellular model for the evaluation of cellular toxicity. The selection of this particular cell line was predicated on its pervasive distribution within the body, its amenability to cultivation, and its notably expeditious doubling time of approximately 24 h. The cells were cultivated in Dulbecco’s Modified Eagle’s Medium (Sigma-Aldrich, St. Louis, MO, USA), with the addition of 10% foetal calf serum (FCS) (Sigma-Aldrich, St. Louis, MO, USA) and 2 mM L-glutamine. The medium was not supplemented with antibiotics. The cultures were then subjected to an incubation process at a temperature of 37 °C in an environment containing 5% carbon dioxide for a duration of seven days.

#### 3.2.2. Cytotoxicity Assessment

The cytotoxicity was conducted by seeding L929 cells into 96-well microtiter plates at a density of 1 × 10^5^ cells/mL. The final volume of the solution was 100 µL per well. The cells were exposed to four different concentrations (10^4^, 10^5^, 10^6^ and 10^7^ M) of the test compounds after a 24-h incubation period under humidified conditions at 37 °C and 5% carbon dioxide. Subsequently, a 2-h tetrazolium-based reaction was initiated by the addition of 10 µL of 2,3-bis(2-methoxy-4-nitro-5-sulfophenyl)-2H-tetrazolium-5-carboxanilide (XTT) labelling reagent to each well. Following the incubation period, a microplate reader (i.e., the Thermo Scientific Microplate Photometer Multiskan FC, USA) was employed to ascertain the degree of absorption at a wavelength of 450 nanometers. For the purpose of establishing a control group, untreated cells were utilized as a negative control, while an additional well containing only culture medium and XTT labeling reagent (10 µL per 100 µL of medium) was employed as a baseline reference.

Stock solutions of complexes **2a** and **2b** were prepared in dimethyl sulfoxide and subsequently diluted to the requisite concentrations for use in the culture medium. The final concentration of dimethyl sulfoxide in all assays did not exceed 2%, which had no detectable effect on the viability of cells or parasites. The solutions were kept in a light-proof container and used within one hour of preparation. All experiments included a solvent control containing 2% dimethyl sulfoxide.

The optical density (OD) values of the treated samples were then compared with those of the negative control group and cell viability was calculated using the following formula: cell viability = [(OD_450_(sample)/OD_450_(negative control)] × 100]. The concentration that reduces cell viability by 50% (CC_50_) was estimated from concentration-response data using log-scale interpolation. To ensure the precision of the experimental measurements, each assay was performed in quadruplicate.

### 3.3. In Vitro Effects of L. major Promastigotes

#### 3.3.1. Parasite Strain and Culture Conditions

The *L. major* strain (MHOM/TR/2013/MANISAPB145) was obtained from the parasite bank at Celal Bayar University. The initial culture was established in the Novy-MacNeal-Nicolson medium. Upon reaching the logarithmic growth phase, the parasites were transferred to the Roswell Park Memorial Institute-1640 medium, which was supplemented with 10% FCS for the purpose of further propagation.

#### 3.3.2. In Vitro Assay for Promastigotes Activity

In vitro experiments were performed using 96-well microplates. Each well was seeded with 100 µL of log-phase promastigotes (5 × 10^5^ promastigotes/mL) together with an equal volume of Roswell Park Memorial Institute-1640 medium that had been supplemented with 10% FBS. The experimental conditions encompassed the administration of varying concentrations (10^−5^, 10^−6^ and 10^−7^ M) of complexes **2a** and **2b**. The cultures were then subjected to an incubation period of 48 h at a temperature of 24 ± 2 °C. Following the incubation period, a tetrazolium-based reaction was initiated for a duration of two hours by adding 10 µL of XTT labeling reagent to each well. Subsequently, a microplate reader (i.e., the Thermo Scientific Microplate Photometer Multiskan FC, Waltham, MA, USA) was employed to ascertain the degree of light absorption at a wavelength of 450 nanometers. A comparison of the absorbance values of the treated samples with those of the control groups was then used to assess metabolic activity and cell viability.

The experimental design incorporated positive and negative controls. The positive control comprised promastigotes that were not exposed to the test compounds, while the negative control contained only culture medium and no parasites. To ascertain that this concentration exerted no discernible effect on the viability of *L. major* promastigotes, a solvent control consisting of a medium containing 2% dimethyl sulfoxide was utilized in parallel. For the in vitro anti-leishmanial assays, the percentage viability of *L. major* promastigotes was plotted relative to the untreated control, with the data presented as a function of log concentration. The estimation of IC_50_ values was conducted through the implementation of log-linear interpolation between adjacent concentrations. The selectivity index was defined as CC_50_(L929)/IC_50_ (*L. major*) to provide an estimate of the therapeutic window. To ensure the precision of the experimental measurements, each assay was performed in quadruplicate.

### 3.4. Experimental Cutaneous Leishmaniosis

#### 3.4.1. Parasites

*L. major* (MHOM/TR/2013/MANISAPB145) amastigotes were obtained from the parasite bank of Celal Bayar University. These amastigotes were propagated through continuous passages in the footpads of BALB/c mice, as outlined in the protocol established by Ozpinar and Polat [55].

#### 3.4.2. Animals

The present study utilized a total of 52 male BALB/c mice. The mice were between four and five weeks of age and weighed approximately 20–25 g. They were maintained under standard laboratory conditions, with a temperature of 22 ± 2 °C, a humidity level of 50–70%, and a 12-h light/dark cycle. The subjects were provided with unrestricted access to drinking water and a standard meal. All experimental procedures were approved in accordance with accepted guidelines for the care of laboratory animals by the Institutional Review and Animal Use Committee of Cumhuriyet University (protocol number: 65202830-050.04.04-58).

#### 3.4.3. Animal Infections and Grouping

A 27.5-gauge needle was utilized to administer 50 µL of sterile, endotoxin-free phosphate-buffered saline (PBS) containing 1 × 10^5^ parasites subcutaneously into the right hind paw pad of each mouse. Three weeks post-infection, the mice were randomly divided into five groups of eight animals each. The study comprised two experimental treatment groups (Groups I and II) and three control groups (one infection-treated group, one infection-untreated group, and one uninfected group; see Table 3).

Prior to the initiation of treatment, the weight and diameter of the lesions were meticulously documented. The test drug was then administered once a week for the duration of the experiment. Prior to administration, complexes **2a** and **2b** were freshly dissolved in 2% dimethyl sulfoxide in sterile PBS. The treatments were administered intralesionally, directly into the infected footpad lesion, at a volume of 50 µL per injection once a week for a period of four weeks. The reference pharmaceutical drug (MA 20 mg/kg/day) was administered subcutaneously in 50 µL of PBS over the course of 20 consecutive days. The control groups received either intralesional 0.9% NaCl once weekly for four weeks, or no infection or treatment.

Throughout the in vivo experimental period, the animals’ general health status, body weight, behavior and local reactions at the injection site were routinely monitored to identify any potential adverse effects of the treatment.

#### 3.4.4. Assessment of Treatment Efficacy

Lesion size: The efficacy of the treatment was determined primarily by measuring changes in lesion size and by conducting a detailed histopathological analysis. The dimensions of the lesions were measured using a digital calliper (RABONE, Geneva, Switzerland) to calculate the area (mm^2^). Additionally, weekly increases in plantar thickness were tracked. A variety of clinical indicators were meticulously monitored to assess the progression of the skin lesions, including erythema, edema, crusting, ulceration, gangrene and autoamputation.

Clinical monitoring: Throughout the in vivo experimental period, animals were routinely monitored on a weekly basis for general health status, changes in body weight, behavioral alterations and local reactions at the injection site, in order to detect any potential adverse effects related to the treatment.

Histopathological analysis: At the conclusion of the treatment regimen, the mice were euthanized. All paw tissues were fixed in 10% formalin. Histological preparations and hematoxylin-eosin (H&E) staining were performed within 12–24 h. Subsequently, each slide was examined under a light microscope (Eclipse E800, Nikon, Tokyo, Japan). The following parameters were assessed: the preservation of subcutaneous tissue structure, the extent of inflammation, the density of amastigotes and the organization of collagen fibers.

For the histopathological evaluation, the entire tissue sections from each animal were initially examined at low magnification in order to assess the overall integrity of the tissue, the extent of inflammatory infiltration, the organization of the collagen and the distribution of the parasites. Based on these predefined histopathological criteria, representative areas were then selected for image acquisition at higher magnifications. The selected images illustrate the general histopathological features observed across the examined sections.

### 3.5. Statistical Analysis

Statistical analyses were performed using GraphPad Prism version 8.0 (GraphPad Software, San Diego, CA, USA) and SPSS version 15.0 (IBM Corp., Armonk, NY, USA). In vitro cytotoxicity and promastigote viability data are presented as mean ± standard error of the mean (SEM), calculated from four technical replicates (quadruplicate wells) per condition obtained within a single experimental run. Comparisons among multiple groups were conducted using one-way analysis of variance (ANOVA), followed by Tukey’s post hoc test for pairwise comparisons. In vivo data (footpad lesion sizes) are expressed as mean ± standard deviation (SD) derived from independent animals in each experimental group. Statistical comparisons between treatment and control groups at corresponding time points were performed using the non-parametric Mann–Whitney U test. In all analyses, a *p* value < 0.05 was considered statistically significant. Asterisks in figures denote significance levels as follows: * *p* < 0.05, ** *p* < 0.01, and *** *p* < 0.001.

### 3.6. Molecular Docking Method

Prior to the molecular docking simulations, the benzimidazolium salts **1a**,**1b** and the silver(I) complexes **2a**,**2b** were optimized using the ORCA package version 6.1.0 [56,57]. The def2-svp def2-svp/j basis set of BP86 was employed for density functional theory optimizations, with tightsf and grid4 restrictions [58]. The RCSB Protein Data Bank was utilized to obtain the crystal structures of LaGP63 (PDB: 1LML [59]), LaARG (PDB: 4ITY [60]), *N*-myristoyltransferase (PDB: 4CGO [61]) and farnesyl diphosphate synthase (PDB: 4K10 [62]) on 25 November 2025. Molecular docking studies were carried out using AutoDockTools 4.2 [63,64]. Polar hydrogens and Kollman charges were assigned during the calculations. All target proteins were prepared in PDBQT format after the removal of water molecules and 150 populations were considered in the Lamarckian genetic algorithm. Ligands were also prepared in pdbqt format using AutoDockTools, with randomized initial conformations and Gasteiger charges applied [65,66]. The coordinates of grid boxes were set as follows: x: 14.000, y: 39.760, z: 14.474 for 1LML; x: 20.078, y: −16.978, z: −5.495 for 4ITY; x: 5.125, y: 47.298, z: 62.080 for 4CGO; x: 37.276, y: 64.267, z: 82.602 for 4K10 with 0.375 spacing value. The grid box sizes were set at 60 × 58 × 54 Å^3^ for 1LML, 70 × 54 × 70 Å^3^ for 4ITY, 60 × 76 × 64 Å^3^ for 4CGO and 62 × 58 × 60 Å^3^ for 4K10. The validation and visualization of all target molecules were performed using Discovery Studio 4.1.0. The validation of the method was achieved through the standard redocking of native ligands. The docking protocol was considered valid when RMSD values were ≤2.0 Å [67].

## 4. Conclusions

In the present study, two silver complexes, chloro[1-methallyl-3-(2,4,6-trimethylbenzyl)-5,6-dimethylbenzimidazole-2-ylidene]silver(I) (**2a**) and chloro[1-methallyl-3-(4-chlorobenzyl)-5,6-dimethylbenzimidazole-2-ylidene]silver(I) (**2b**), were investigated for the treatment of cutaneous leishmaniasis (CL). The results of our study provide compelling evidence for the therapeutic potential of silver(I) complexes in the treatment of CL. These complexes exhibited substantial efficacy in diminishing lesion size in a mouse model, underscoring their potential as promising candidates for further research and development as alternative therapeutic options. The efficacy of our complexes, complex **2b** demonstrates moderately superior efficiency in comparison to its homologue **2a** and has been demonstrated to exceed that of analogous chloro[1-methallyl-3-(arylmethyl)-5,6-dimethylbenzimidazole-2-ylidene]silver(I) complexes that have been the subject of prior investigations [39].

The silver(I) complexes **2a**,**2b** and their benzimidazolium salt precursors were also analyzed by molecular docking against LaGP63, LaARG, *N*-myristoyltransferase and Farnesyl pyrophosphate synthase to better understand anti-leishmanial activity. A comparison of the docking performance of the silver(I) complexes and the salts revealed that the former exhibited superior activity. Complex **2a** demonstrates marginally superior binding affinities in comparison to its homologue **2b**.

Further research is necessary to elucidate the precise mechanisms underlying the therapeutic effects and optimize treatment protocols. However, the results of this study highlight the importance of diversifying the therapeutic arsenal against CL. The silver(I) complexes **2a**,**2b** represent a significant advancement in this field, offering a promising avenue for the development and enhancement of treatment strategies for this debilitating disease.

## Figures and Tables

**Table 1:** The size of the footpad lesions (mm^2^) and the statistical evaluation between the groups studied during the experimental period ^1^.

Weeks Post Infection	Group I Complex 2a (10^−5^ M)	Group II Complex 2b (10^−5^ M)	Group III MA ^2^ (20 mg/kg/day)	Group IV NaCl ^2^ (0.9%)
1st	55.4 ± 9.9	54.3 ± 1.3	55.2 ± 1.7	56.6 ± 3.0
Sig. with	/	/	/	/
2nd	32.4 ± 8.4	31.2 ± 4.3	46.3 ± 8.5	59.5 ± 3.2
Sig. with	Group IV *	Group IV *	/	Group I *, II *
3rd	27.8 ± 3.6	22.6 ± 6.6	44.7 ± 3.4	63.1 ± 7.7
Sig. with	Group III *, IV **	Group III **, IV **	Group I *, II *	Group I **, II **
4th	23.4 ± 3.6	16.6 ± 2.6	37.9 ± 1.8	70.7 ± 4.7
Sig. with	Group III **, IV ***	Group III **, IV ***	Group I **, II **	Group I ***, II ***
5th	20.9 ± 3.1	11.2 ± 4.6	33.2 ± 3.6	68.8 ± 1.7
Sig. with	Group III **, IV ***	Group III **, IV ***	Group I **, II **, IV **	Group I ***, II ***, III **

^1^ Data were described using mean ± SD * Sig. *p* < 0.05, ** Sig. *p* < 0.01, *** Sig. *p* < 0.001. ^2^ MA: meglumine antimoniate; ^2^ NaCl: sodium chloride.

**Table 2:** Binding affinity, interacting amino acids and interaction types between salts **1a**,**1b** and complexes **2a**,**2b** against LaGP63, LaARG, *N*-myristoyltransferase and farnesyl diphosphate synthase.

Drugs	BA * (kcal/mol)	Amino Acids Residue
LaGP63		
**1a**	−5.40	Ala349 (H-bond), Glu265 (π-interaction), Val223, Leu224, Leu257, Val261, His264, His268, His334, Ala348 (alkylic interactions), Gly222, Ala225, Pro347, Phe451 (van der Waals interactions)
**1b**	−6.23	His264, Glu265 (π-interactions), Leu224, Arg260, Val261, His268, His334, Ala349 (alkylic interactions), Gly222, Val223, Ala225, Glu343, Ala346, Pro347, Ala348, Ala350, Gly352 (van der Waals interactions)
**2a**	−6.98	His264, Glu265 (π-interactions), His224, Leu257, His268, His334, Ala348, Ala349, Phe451 (alkylic interactions), Val223, Ala225, Val261, Ser330, Ala346, Pro347 (van der Waals interactions)
**2b**	−6.49	His264, Ala349 (π-interactions), Leu224, Val261, His268, His334, Ala348, Phe451 (alkylic interactions), Gly222, Arg260, Glu265, Gly329, Ser330, Glu343, Leu344, Ala346, Pro347, Ala350, Gly352 (van der Waals interactions)
LaARG		
**1a**	−3.27	His154, Thr257 (π-interactions), Pro27, His114, His139, Ala140 (alkylic interactions), His28, Asp141, Ile142, Ser150, Asn152, Gly155, Ala192, Val193, Asp194, Glu197, Asp243, Asp245, Glu288 (van der Waals interactions)
**1b**	−3.77	Ser150, Thr257 (H-bonds), His154 (π-interactions), His139, Ala192 (alkylic interactions), His28, Asp137, Asp141, Asn143, Asn152, Gly155, Val193, Asp194, Glu197, Asp243, Asp245, Gly256, Glu288 (van der Waals interactions)
**2a**	−4.47	His139, Thr257 (π-interactions), Pro27, His28, His154, Ala192, Pro258 (alkylic interactions), Gln26, Ala140, Asp141, Gly155, Val193, Asp194, Glu197, Gly256 (van der Waals interactions)
**2b**	−4.27	His28, His139, Ala140 (alkylic interactions), Gln26, Pro27, Asp141, Ile142, Asn143, Asn152, Gly155, Glu197, Gly256, Thr257 (van der Waals interactions)
*N*-Myristoyltransferase		
**1a**	−7.97	Asn167, Thr203, Tyr217 (H-bonds), Tyr80 (π-interaction), Val81, Phe90, Ile166, Leu169, Ala204, Ile328, Tyr345, Leu399, Met420 (alkylic interactions), Tyr92, Phe168, Tyr202, Gly205, Leu421 (van der Waals interactions)
**1b**	−7.76	Thr80, Ile166, Thr203 (H-bonds), Ala204, Leu421 (π-interactions), Val81, Phe90, Leu169, Tyr202, Tyr217, Tyr345, Leu399 (alkylic interactions), Tyr92, Ile126, Asn167, Phe168, Gly205, Met420 (van der Waals interactions)
**2a**	−8.27	Tyr80, Tyr92, Leu421 (H-bonds), Val81, Phe90, Tyr217, Ile328, Tyr345, Leu399, Met420 (alkylic interactions), Asn167, Phe168, Leu169, Thr203, Ala204, Gly205, Gly397, His398 (van der Waals interactions)
**2b**	−8.17	Thr203 (H-bond), Val81, Ile166, Leu169, Tyr202, Ala204, Tyr217, Leu399, Met420 (alkylic interactions), Tyr80, Tyr92, Asn167, Phe168, Gly205, His398, Leu421 (van der Waals interactions)
Farnesyl diphosphate synthase		
**1a**	−5.06	Thr208, Gln247 (H-bonds), Arg51, Lys207, Phe246 (π-interactions), Phe94, Leu95, Tyr211 (alkylic interactions), Lys48, Gln91, Asp98, Asp99, Arg107, Thhr163, Gln167, Asp250 (van der Waals interactions)
**1b**	−5.16	Thr208, Gln247 (H-bonds), Phe94, Leu95, Lys207, Tyr211, Phe246 (alkylic interactions), Lys48, Arg51, Gln91, Asp98, Arg107, Thr163, Gln167, Asp250 (van der Waals interactions)
**2a**	−5.35	Thr208, Tyr211, Gln247 (H-bonds), Arg51, Phe246 (π-interactions), Phe94, Leu95 (alkylic interactions), Lys48, Gln91, Asp98, Arg107, Thr163, Gln167, Asp250, Lys264 (van der Waals interactions)
**2b**	−5.20	Thr208, Tyr211, Gln247 (H-bonds), Phe94, Leu95, Lys207, Phe246 (alkylic interactions), Lys48, Arg51, Gln91, Asp98, Arg107, Thr163, Gln167, Asp250, Lys264, Lys273 (van der Waals interactions)

* BA: Binding affinity.

**Table 3:** Test groups, treatment doses, method of treatment and number of animals used.

Groups	Treatment Doses	Treatment Method	Animal Number
Group I	10^−5^ M once a week for **2a**	Intralesional—4 weeks	8
Group II	10^−5^ M once a week for **2b**	Intralesional—4 weeks	8
Group III (control-infected, treated group)	Meglumine antimoniate (Glucantime^®^) (20 mg/kg/day)	Subcutaneously—20 days	8
Group IV (control-infected, non-treated group)	0.9% (*w*/*v*)-NaCl	Intralesional—once a week	8
Group V (Non-infected group)	/	/	4

## Data Availability

The original contributions presented in the study are included in the article/Supplementary Materials, further inquiries can be directed to the corresponding authors.

## Supplementary Materials

The following supporting information can be downloaded at: https://www.mdpi.com/article/10.3390/ph19030356/s1, Figure S1: FT-IR spectrum of **2a**; Figure S2: ^1^H NMR spectrum of **2a**; Figure S3: ^13^C{^1^H} NMR spectrum of **2a**; Figure S4: FT-IR spectrum of **2b**; Figure S5: ^1^H NMR spectrum of **2b**; Figure S6: ^13^C{^1^H} NMR spectrum of **2b**; Figure S7: Interaction details and residues of **2a** against LaGP63; Figure S8: Interaction details and residues of **2b** against LaGP63; Figure S9: Interaction details and residues of **1a** against LaARG; Figure S10: Interaction details and residues of **1b** against LaARG; Figure S11: Interaction details and residues of **2a** against LaARG; Figure S12: Interaction details and residues of **2b** against LaARG; Figure S13: Interaction details and residues of **1a** against *N*-myristoyltransferase; Figure S14: Interaction details and residues of **1b** against *N*-myristoyltransferase; Figure S15: Interaction details and residues of **2b** against *N*-myristoyltransferase; Figure S16: Interaction details and residues of **2b** against *N*-myristoyltransferase; Figure S17: Interaction details and residues of **1a** against farnesyl diphosphate synthase; Figure S18: Interaction details and residues of **1b** against farnesyl diphosphate synthase; Figure S19: Interaction details and residues of **2a** against farnesyl diphosphate synthase; Figure S20: Interaction details and residues of **2b** against farnesyl diphosphate synthase; Figure S21: Interaction details and residues of **1b** against farnesyl diphosphate synthase; Figure S22: Interaction details and residues of **2a** against farnesyl diphosphate synthase; Figure S23: Interaction details and residues of **2b** against farnesyl diphosphate synthase.

## Abbreviations

The following abbreviations are used in this manuscript:

CL	cutaneous leishmaniasis
NHC	*N*-heterocyclic carbenes
WHO	World Health Organization
NMR	nuclear magnetic resonance
XTT	2,3-bis(2-methoxy-4-nitro-5-sulfophenyl)-2H-tetrazolium-5-carboxanilide
OD	optical density
FCS	foetal calf serum
PBS	endotoxin-free phosphate-buffered saline
MA	meglumine antimoniate
