# Supplementary material for: Silver-N-Heterocyclic Complexes Against Leishmania major: In Vitro, In Vivo and In Silico Therapeutic Activities
Source: Pharmaceuticals (Basel). 2026 Feb 25;19(3):356. doi: 10.3390/ph19030356 (PMC13028932; doi:10.3390/ph19030356)
Supplement: Supplementary file 1 [file pharmaceuticals-19-00356-s001.zip › pharmaceuticals-4148282-supplementary.pdf]

Silver-*N*-heterocyclic complexes against *Leishmania major: In vitro*, *in vivo,* and *in-silico* therapeutic activities

Neslihan Şahin [1,2], Zübeyda Akın Polat [3], Derya Gül Gülpınar [3], Ahmet Duran Ataş [3], Elvan Üstün [4,*], İsmail Özdemir [5] and David Sémeril [6,*]

1 Malatya Turgut Özal University, Faculty of Engineering and Natural Sciences, Department of Engineering Basic Sciences, 44900 Malatya, Türkiye
2 Cumhuriyet University, Faculty of Education, Department of Science Education, 58040 Sivas, Türkiye
3 Cumhuriyet University, Department of Medical Parasitology, School of Medicine, 58140 Sivas, Türkiye
4 Ordu University, Faculty of Art and Science, Department of Chemistry, 52200 Ordu, Türkiye
5 İnönü University, Faculty of Art and Science, Department of Chemistry, 44280 Malatya, Türkiye
6 Strasbourg University, Synthèse Organométallique et Catalyse, UMR-CNRS 7177, 67008 Strasbourg, France

Supporting Information

Content		Page
**Figure S1**	FT-IR spectrum of **2a**	3
**Figure S2**	^{1}H NMR spectrum of **2a**	3
**Figure S3**	^{13}C{^{1}H} NMR spectrum of **2a**	4
**Figure S4**	FT-IR spectrum of **2b**	4
**Figure S5**	^{1}H NMR spectrum of **2b**	5
**Figure S6**	^{13}C{^{1}H} NMR spectrum of **2b**	6
**Figure S7**	^{1}H NMR spectra of **2b** at different time intervals	6
**Figure S8**	Interaction details and residues of **1a** against LaGP63	8
**Figure S9**	Interaction details and residues of **1b** against LaGP63	8
**Figure S10**	Interaction details and residues of **2a** against LaGP63	9
**Figure S11**	Interaction details and residues of **2b** against LaGP63	9
**Figure S12**	Interaction details and residues of **1a** against LaARG	10
**Figure S13**	Interaction details and residues of **1b** against LaARG	10
**Figure S14**	Interaction details and residues of **2a** against LaARG	11
**Figure S15**	Interaction details and residues of **2b** against LaARG	11
**Figure S16**	Interaction details and residues of **1a** against *N*-myristoyltransferase	12
**Figure S17**	Interaction details and residues of **1b** against *N*-myristoyltransferase	12
**Figure S18**	Interaction details and residues of **2b** against *N*-myristoyltransferase	13
**Figure S19**	Interaction details and residues of **2b** against *N*-myristoyltransferase	13
**Figure S20**	Interaction details and residues of **1a** against farnesyl diphosphate synthase	14
**Figure S21**	Interaction details and residues of **1b** against farnesyl diphosphate synthase	14
**Figure S22**	Interaction details and residues of **2a** against farnesyl diphosphate synthase	15
**Figure S23**	Interaction details and residues of **2b** against farnesyl diphosphate synthase	15

Chloro[1-methallyl-3-(2,4,6-trimethylbenzyl)-5,6-dimethylbenzimidazole-2-ylidene]silver(I) (2a)

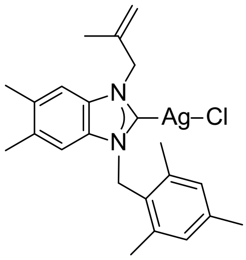

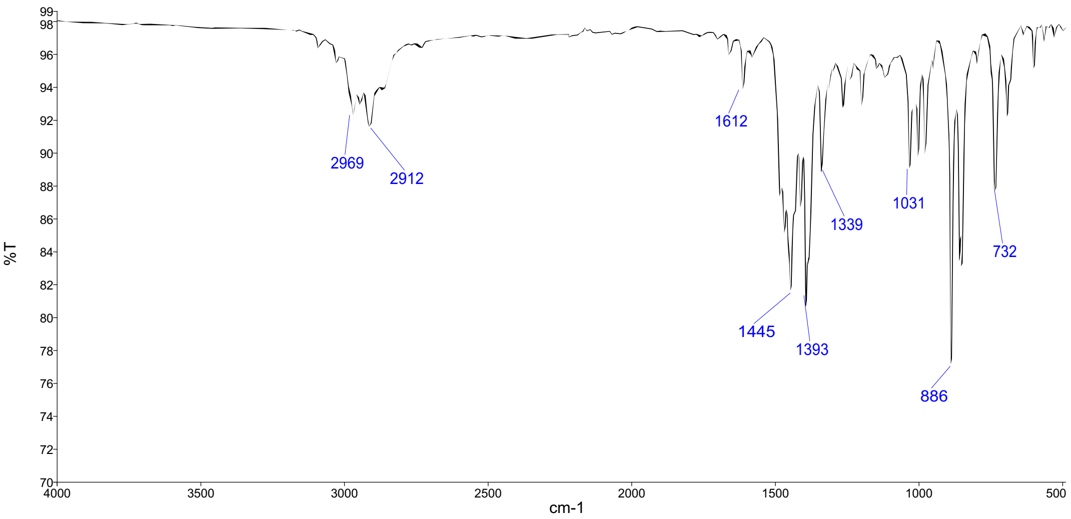

Figure S1. FT-IR spectrum of **2a**

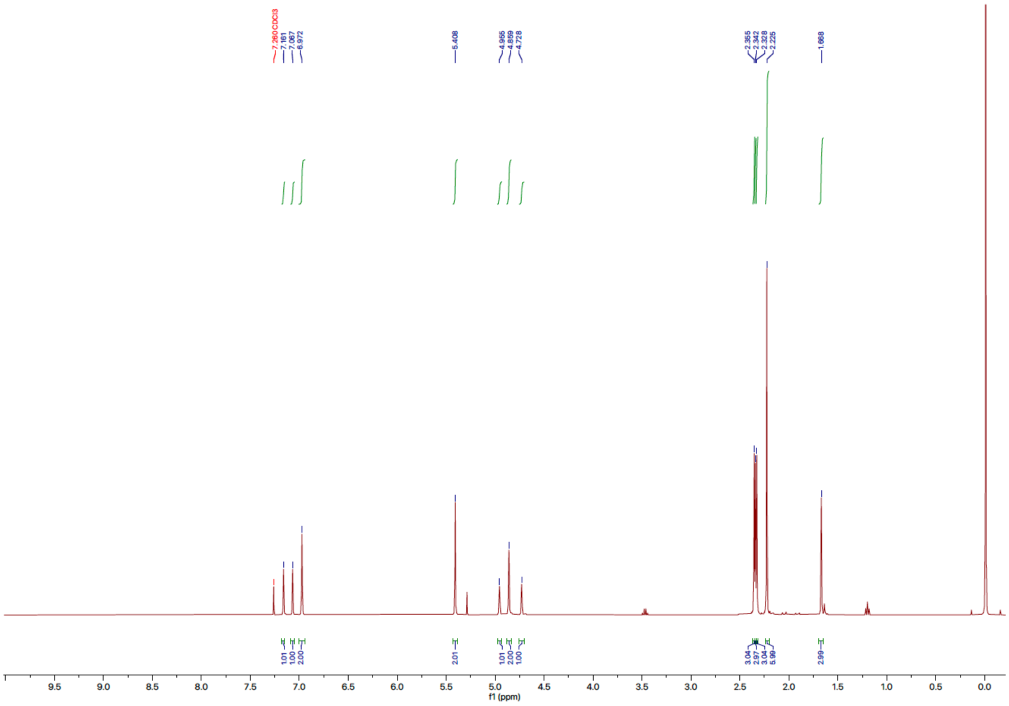

Figure S2. ^{1}H NMR spectrum ($CDCl_3$) of **2a**

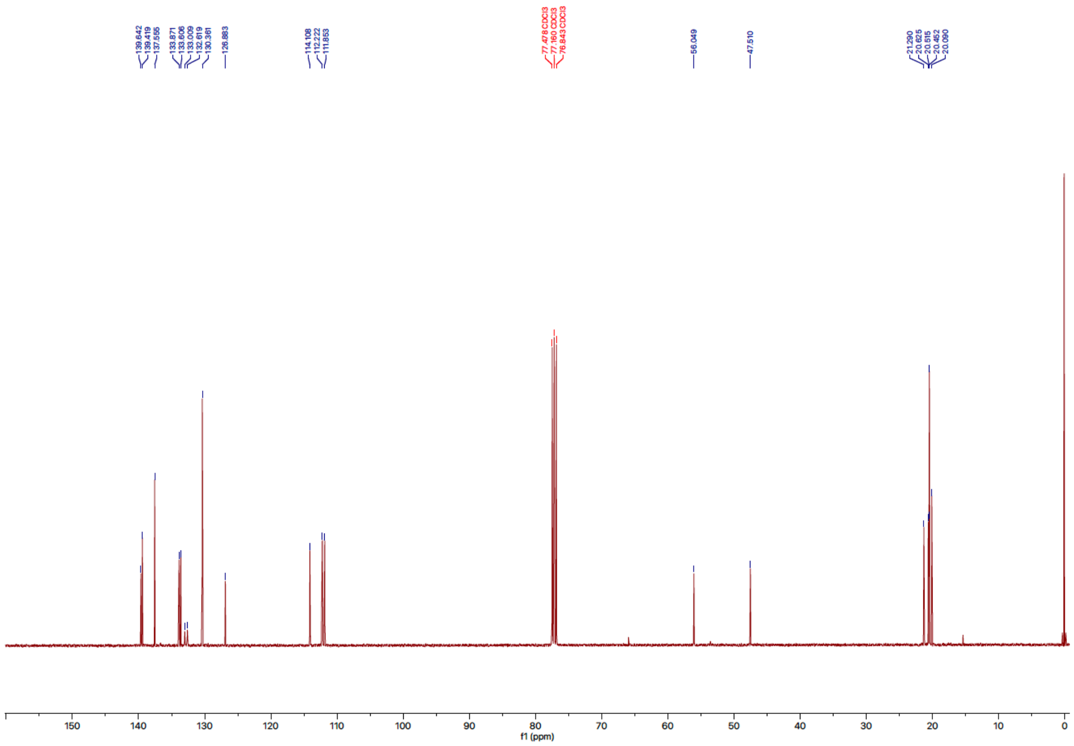

Figure S3. $^{13}C\{^{1}H\}$ NMR spectrum ($CDCl_3$) of **2a**

Chloro[1-methallyl-3-(4-chlorobenzyl)-5,6-dimethylbenzimidazole-2-ylidene]silver(I) (2b)

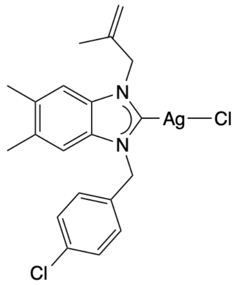

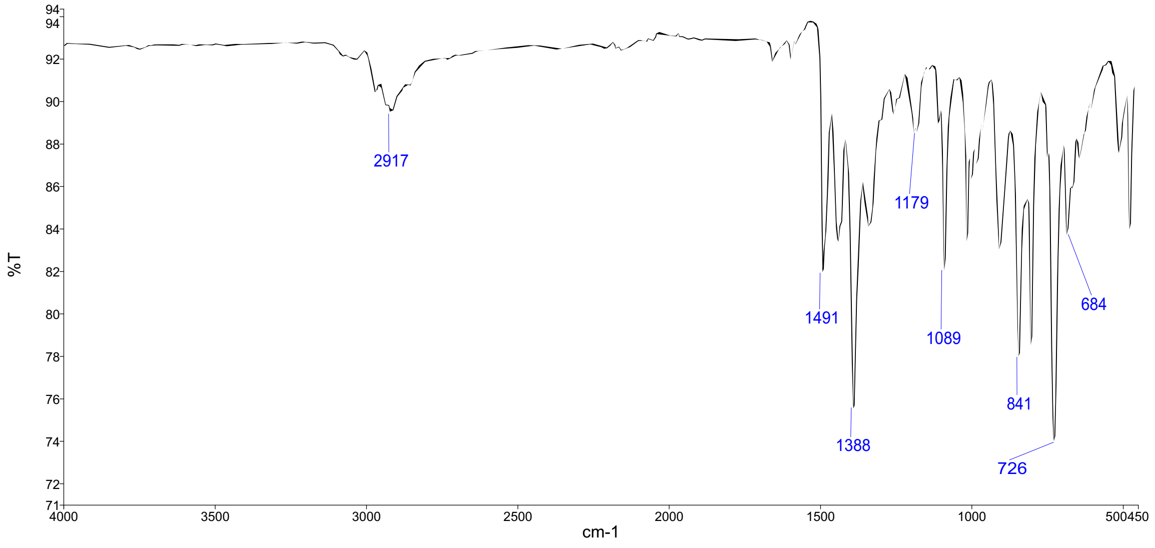

Figure S4. FT-IR spectrum of **2b**

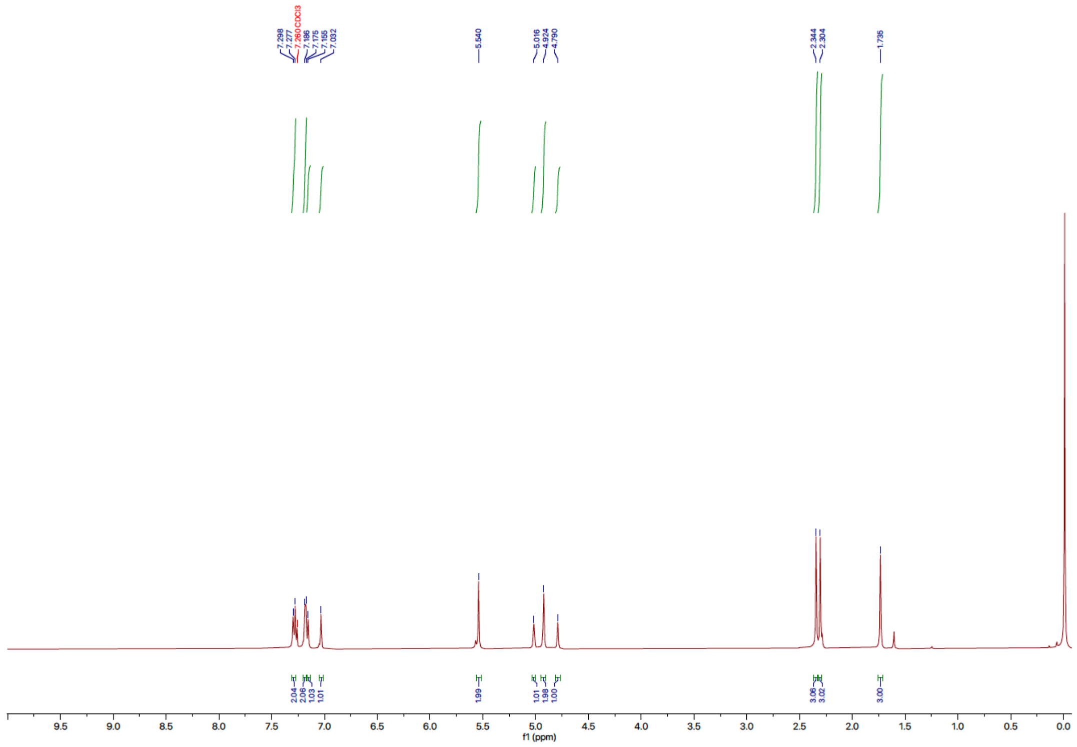

Figure S5. ^{1}H NMR spectrum ($CDCl_3$) of **2b**

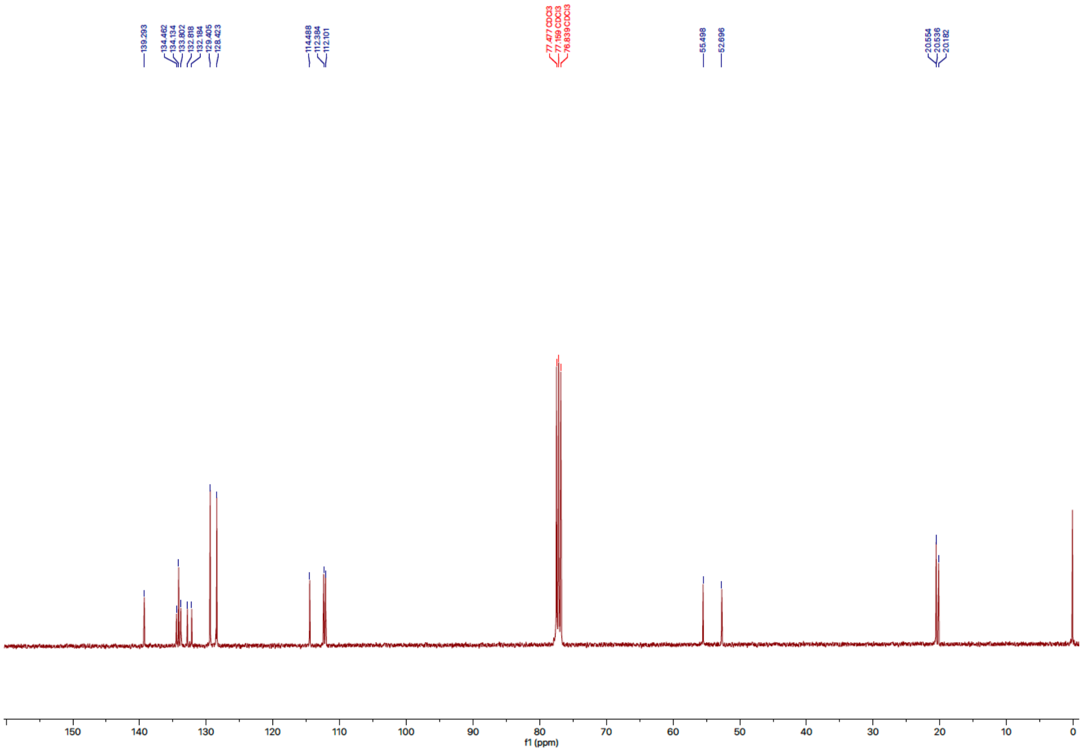

Figure S6. $^{13}C\{^{1}H\}$ NMR spectrum ($CDCl_3$) of **2b**

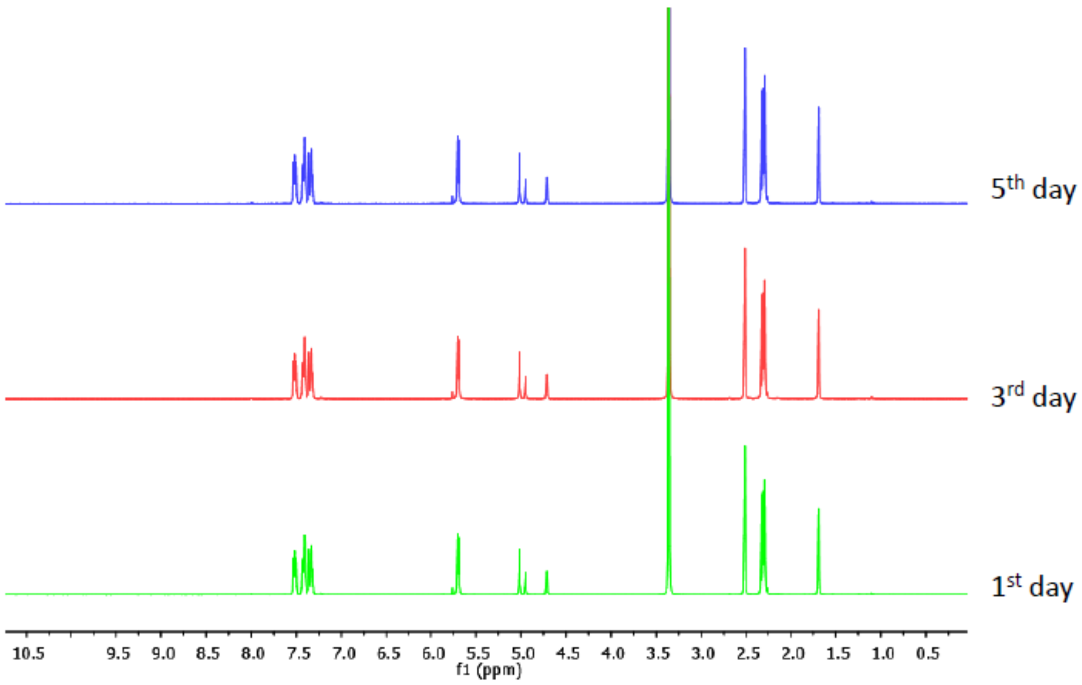

Figure S7. ^{1}H NMR spectra of **2b** at different time intervals

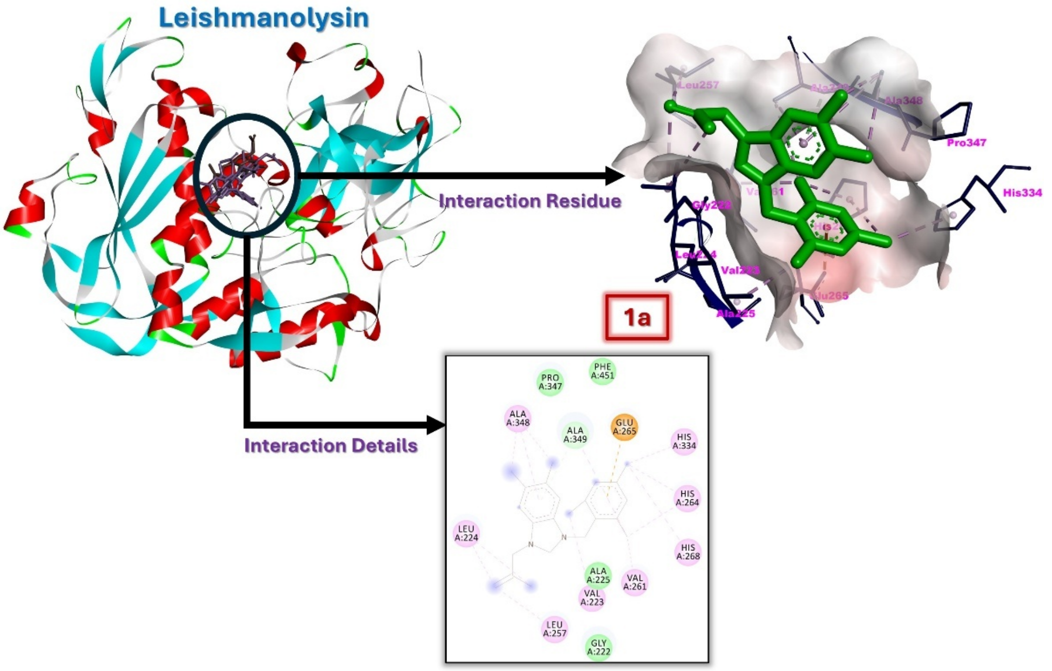

Figure S8. Interaction details and residues of **1a** against LaGP63

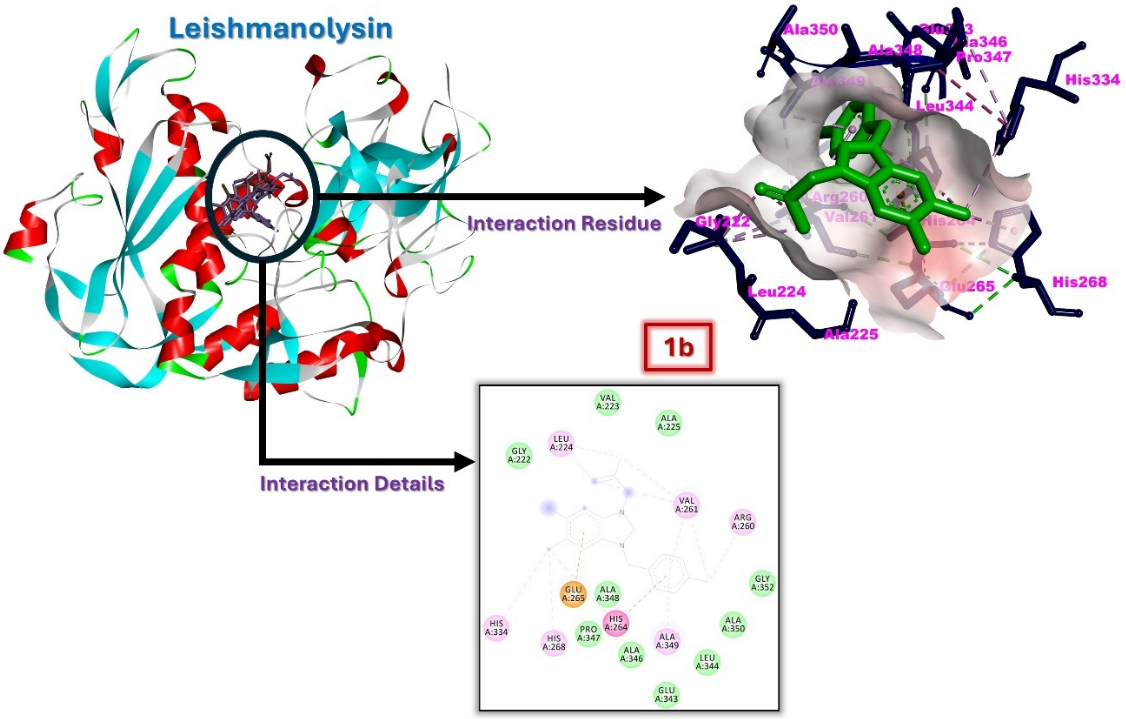

Figure S9. Interaction details and residues of **1b** against LaGP63

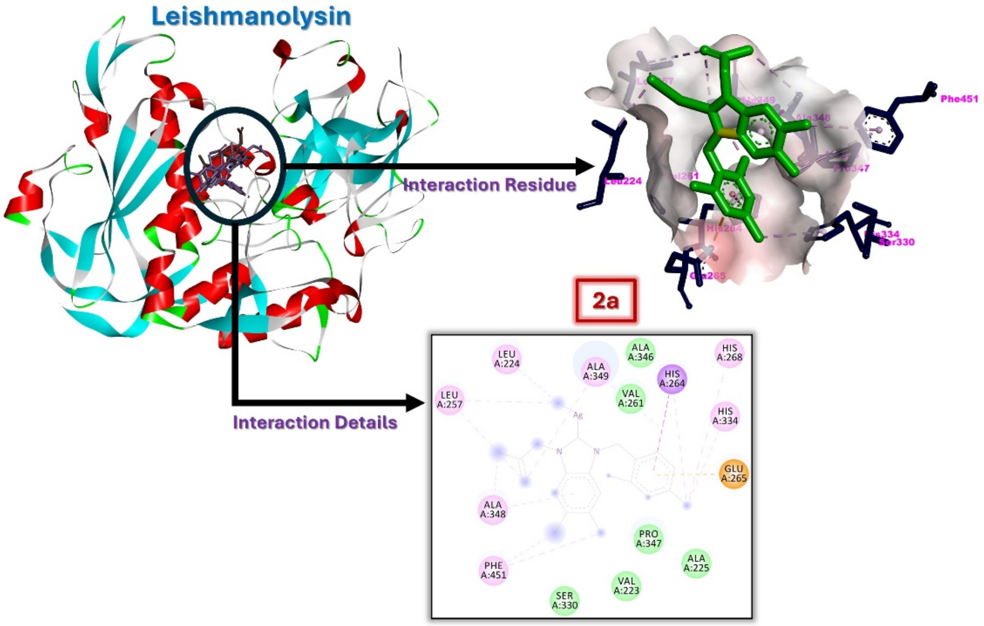

Figure 10. Interaction details and residues of **2a** against LaGP63

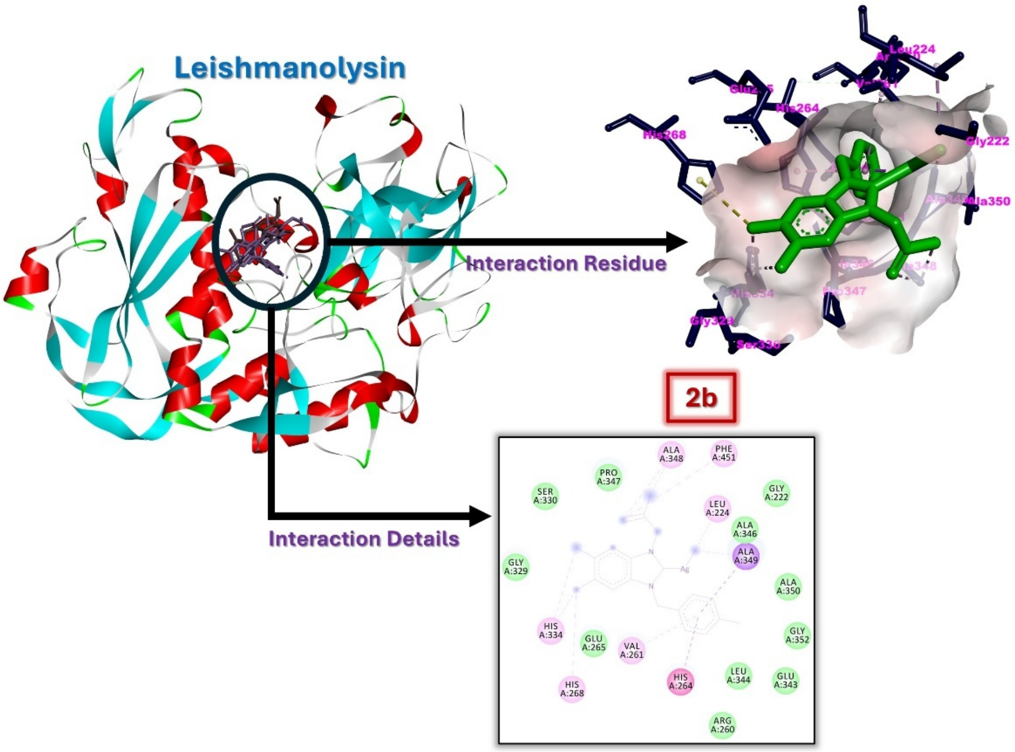

Figure S11. Interaction details and residues of **2b** against LaGP63

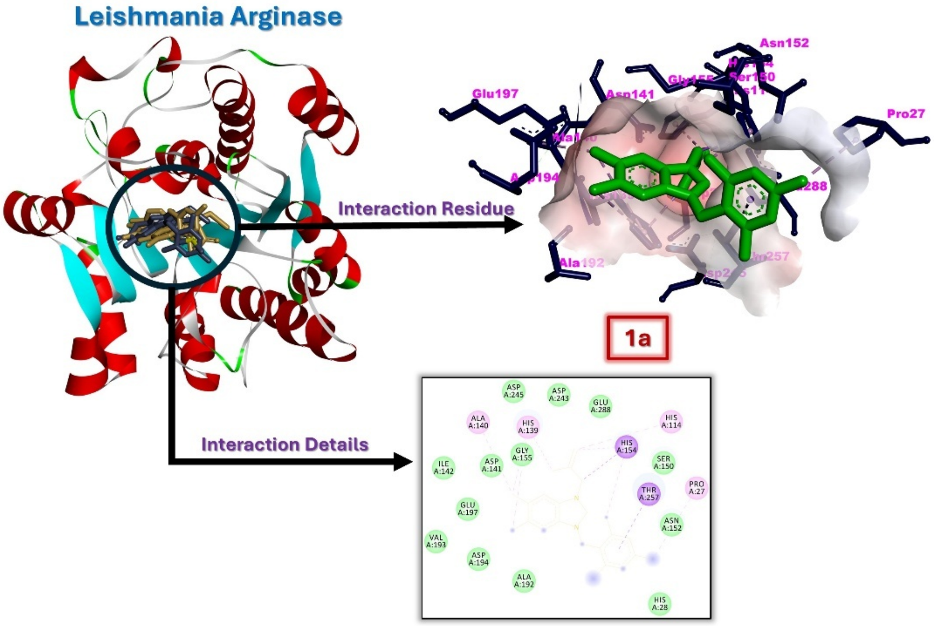

Figure S12. Interaction details and residues of **1a** against LaARG

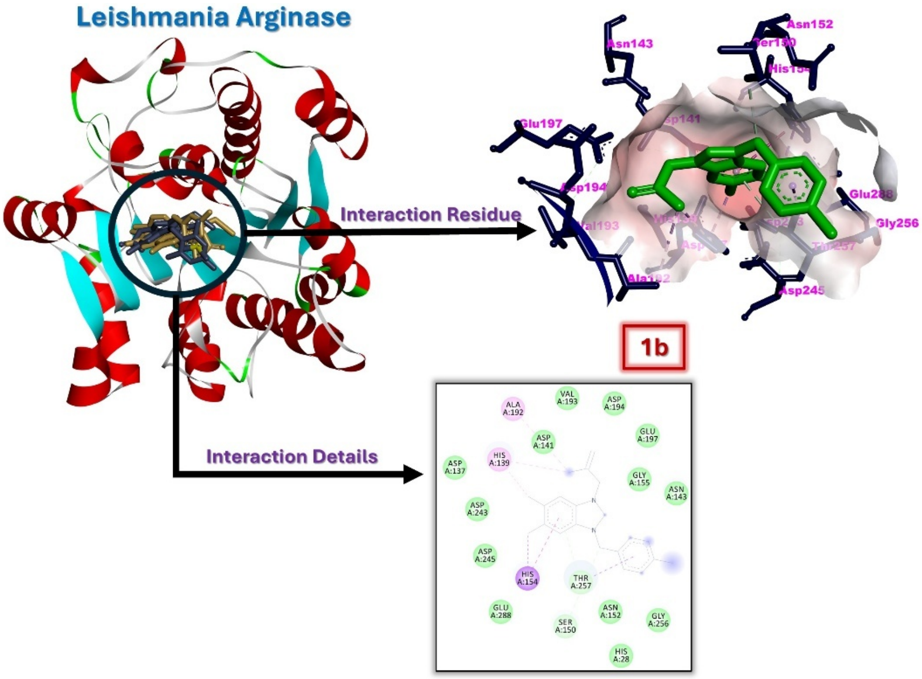

Figure S13. Interaction details and residues of **1b** against LaARG

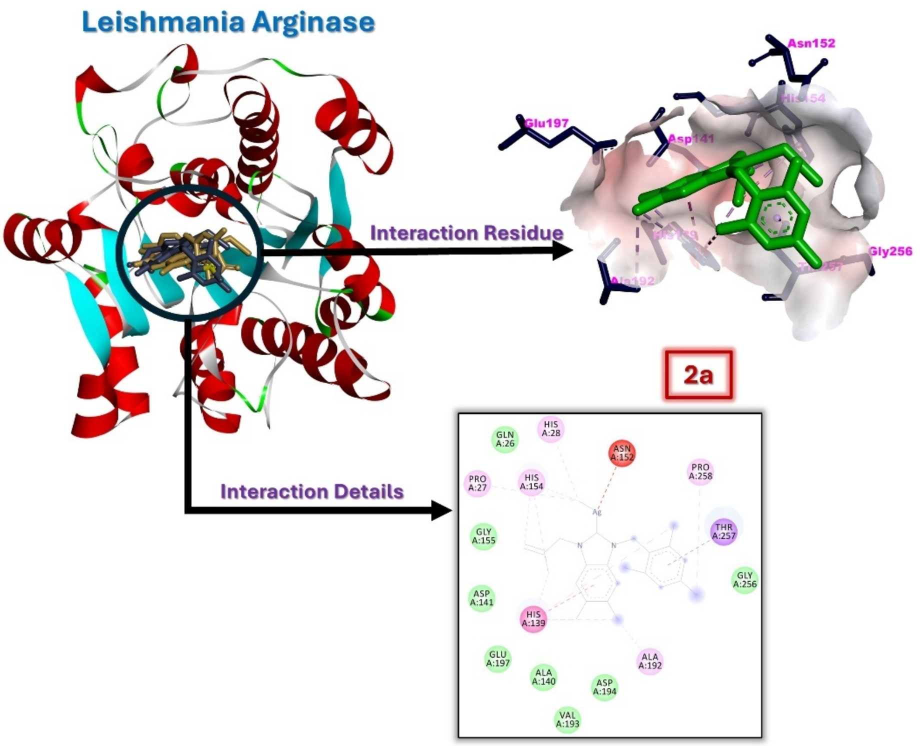

Figure S14. Interaction details and residues of **2b** against LaARG

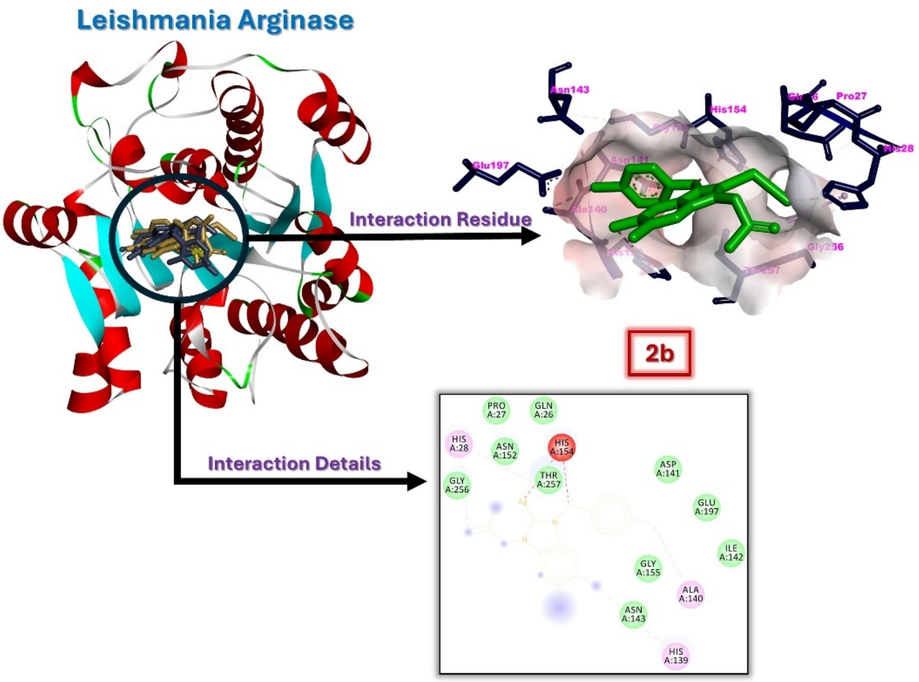

Figure S15. Interaction details and residues of **2b** against LaARG

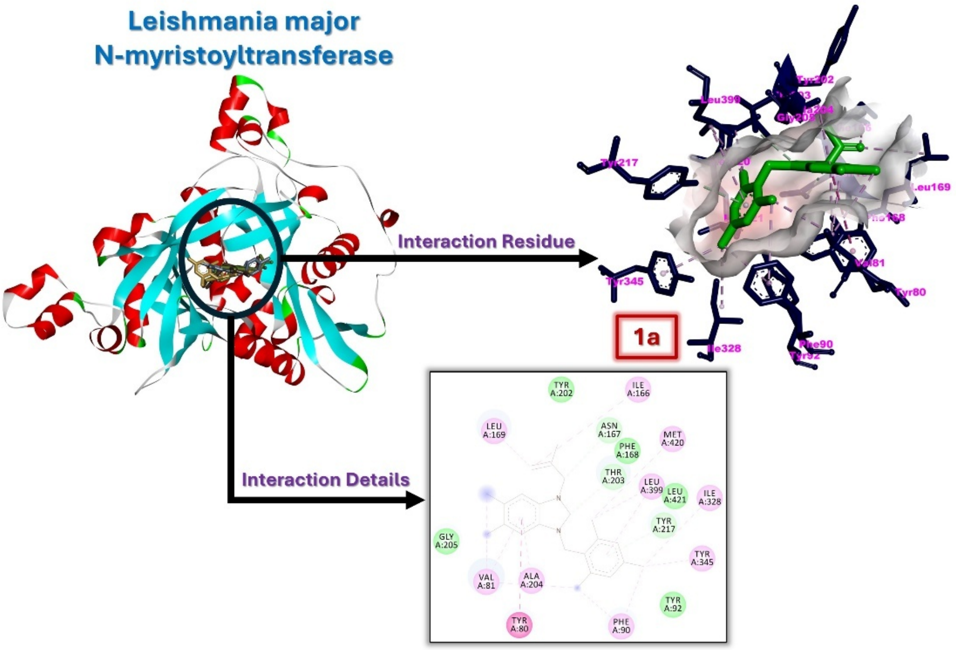

Figure S16. Interaction details and residues of **1a** against *N*-myristoyltransferase

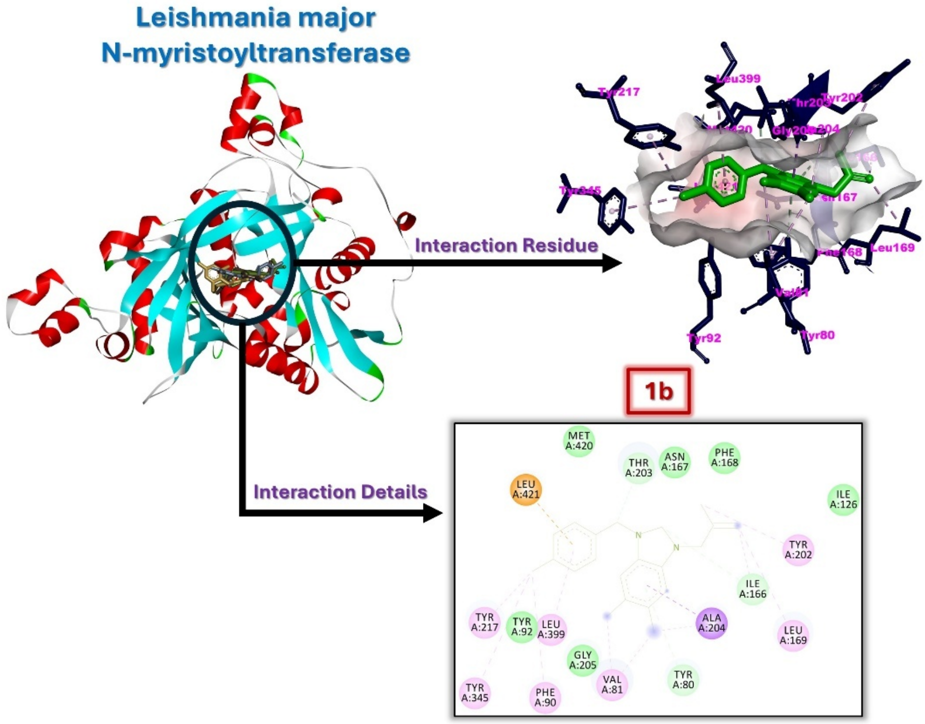

Figure S17. Interaction details and residues of **1b** against *N*-myristoyltransferase

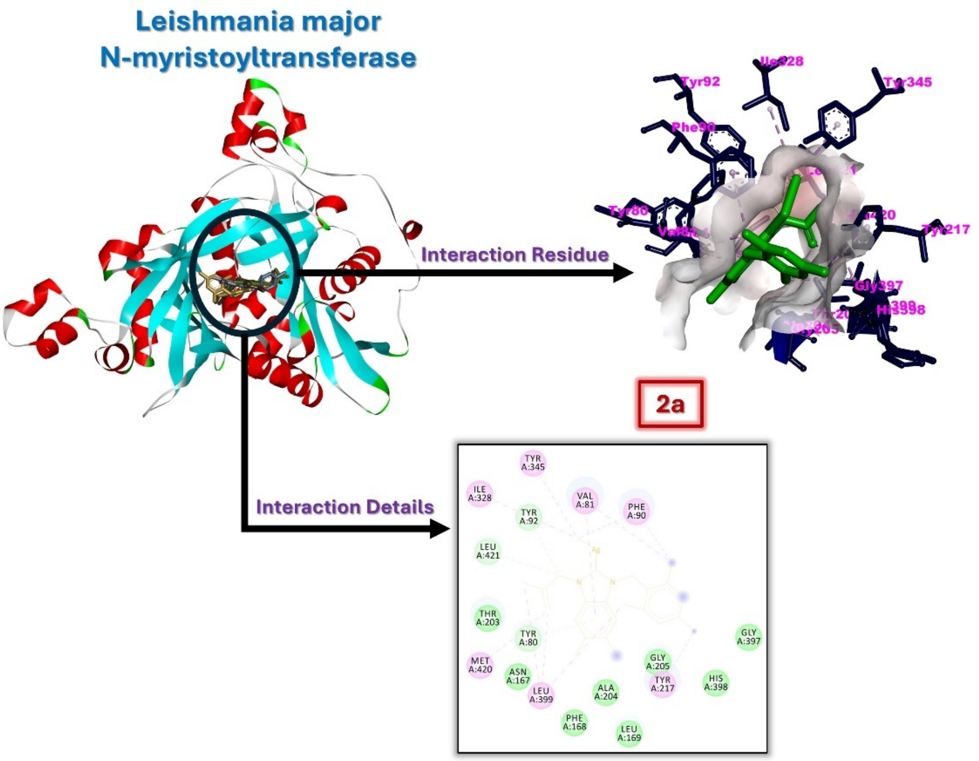

Figure S18. Interaction details and residues of **2a** against *N*-myristoyltransferase

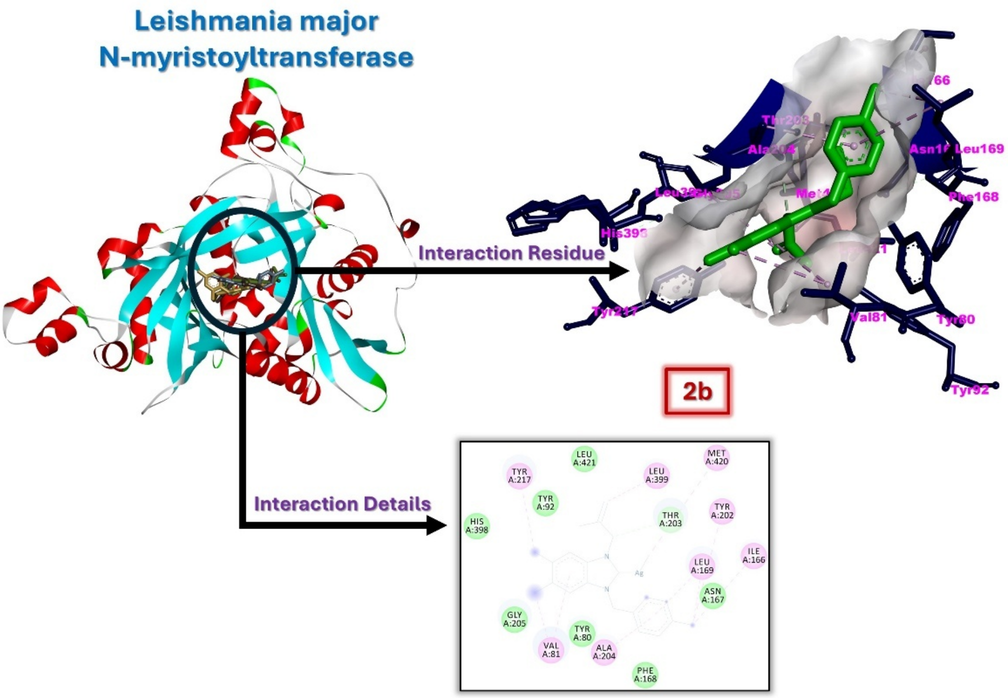

Figure S19. Interaction details and residues of **2b** against *N*-myristoyltransferase

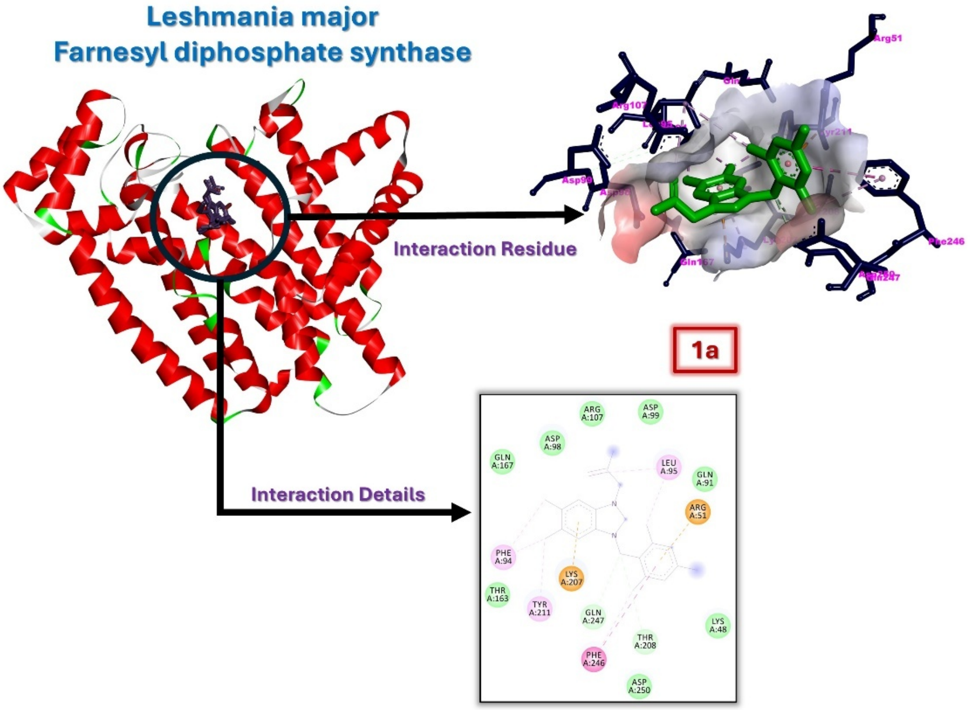

Figure S20. Interaction details and residues of **1a** against farnesyl diphosphate synthase

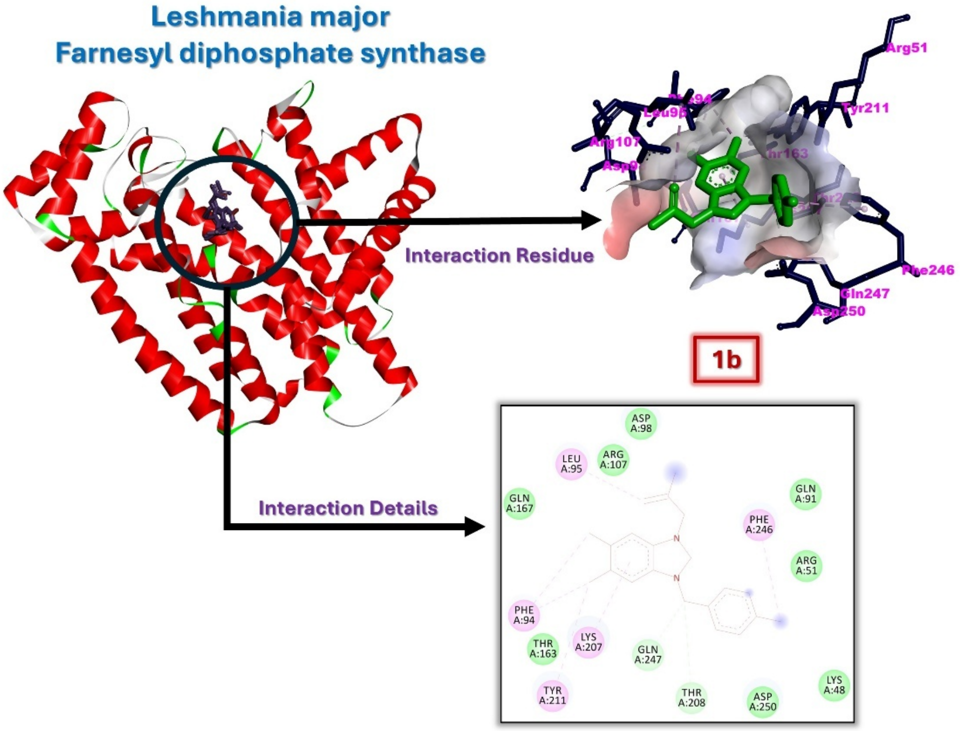

Figure S21. Interaction details and residues of **1b** against farnesyl diphosphate synthase

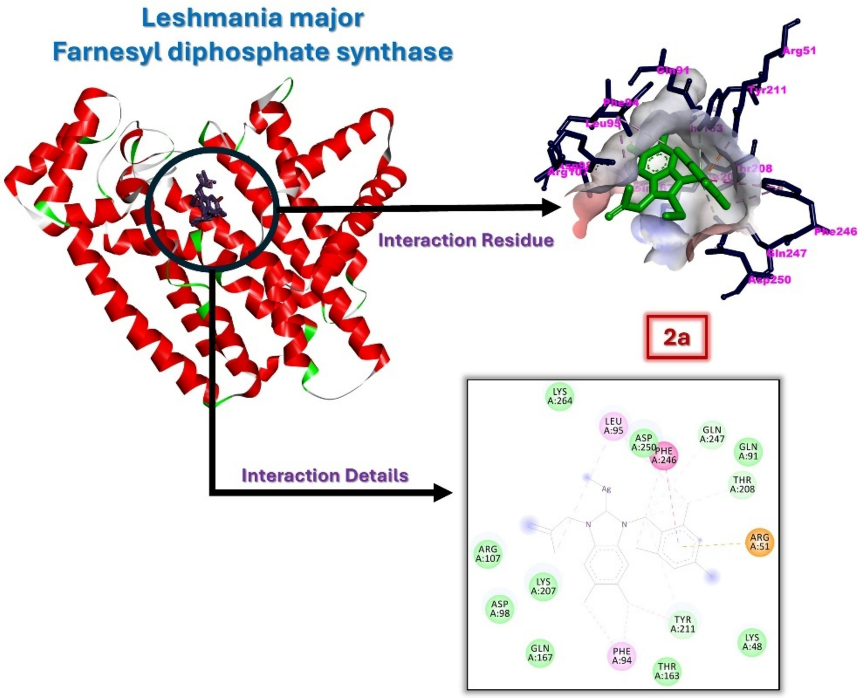

Figure S22. Interaction details and residues of **2a** against farnesyl diphosphate synthase

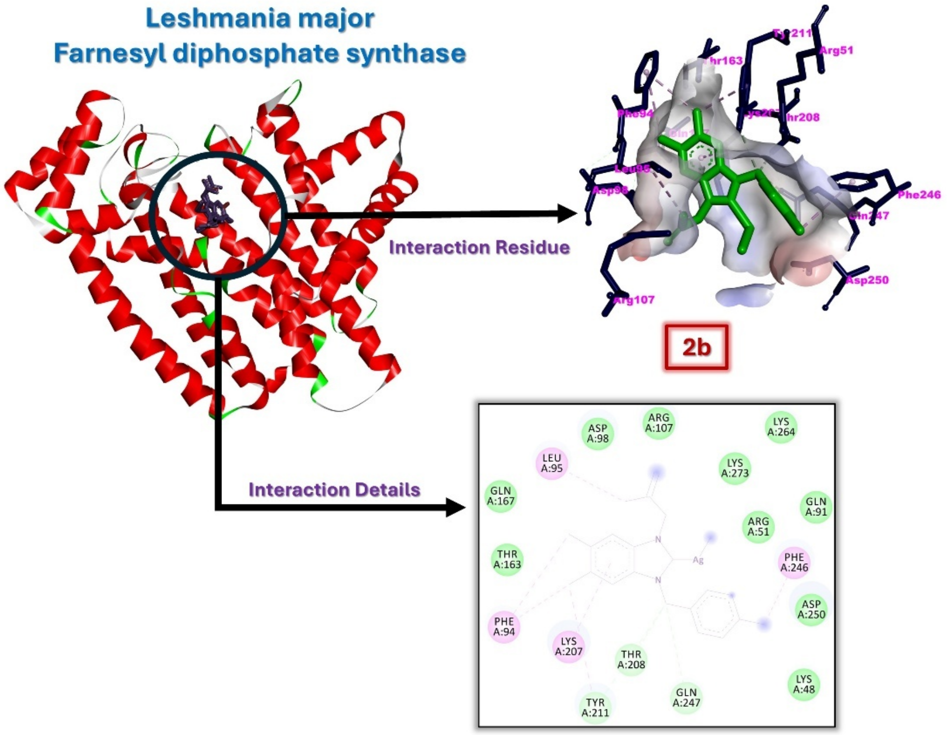

Figure S23. Interaction details and residues of **2b** against farnesyl diphosphate synthase
